# Oxidative Stress in Human Pathology and Aging: Molecular Mechanisms and Perspectives

**DOI:** 10.3390/cells11030552

**Published:** 2022-02-05

**Authors:** Younis Ahmad Hajam, Raksha Rani, Shahid Yousuf Ganie, Tariq Ahmad Sheikh, Darakhshan Javaid, Syed Sanober Qadri, Sreepoorna Pramodh, Ahmad Alsulimani, Mustfa F. Alkhanani, Steve Harakeh, Arif Hussain, Shafiul Haque, Mohd Salim Reshi

**Affiliations:** 1 Department of Biosciences, Division Zoology, Career Point University, Hamirpur 176041, India; younismajeed64@gmail.com (Y.A.H.); rakshasharma429@gmail.com (R.R.); 2 Toxicology and Pharmocology Laboratory, Department of Zoology, School of Biosciences and Biotechnology, Baba Ghulam Shah Badshah University, Rajouri 185234, India; unikshahid@gmail.com (S.Y.G.); sheikhtariq669@gmail.com (T.A.S.); djaved383@gmail.com (D.J.); sanoberqadrisyed@gmail.com (S.S.Q.); 3 Department of Life and Environmental Sciences, College Natural & Health Sciences, Zayed University, Dubai P.O. Box 19282, United Arab Emirates; sreepoorna.unni@zu.ac.ae; 4 Medical Laboratory Technology Department, College of Applied Medical Sciences, Jazan University, Jazan 45142, Saudi Arabia; aalsulimani@jazanu.edu.sa; 5 Emergency Service Department, College of Applied Sciences, AlMaarefa University, Riyadh 11597, Saudi Arabia; mkhanani@mcst.edu.sa; 6 King Fahd Medical Research Center, and Yousef Abdullatif Jameel Chair of Prophetic Medicine Application, King Abdulaziz University, Jeddah 21589, Saudi Arabia; sharakeh@gmail.com; 7 School of Life Sciences, Manipal Academy of Higher Education, Dubai P.O. Box 345050, United Arab Emirates; 8 Research and Scientific Studies Unit, College of Nursing and Allied Health Sciences, Jazan University, Jazan 45142, Saudi Arabia; 9 Bursa Uludağ University Faculty of Medicine, Görükle Campus, Nilüfer 16059, Bursa, Turkey

**Keywords:** oxidative stress, reactive oxygen species, pathologies, mechanisms, aging

## Abstract

Reactive oxygen and nitrogen species (RONS) are generated through various endogenous and exogenous processes; however, they are neutralized by enzymatic and non-enzymatic antioxidants. An imbalance between the generation and neutralization of oxidants results in the progression to oxidative stress (OS), which in turn gives rise to various diseases, disorders and aging. The characteristics of aging include the progressive loss of function in tissues and organs. The theory of aging explains that age-related functional losses are due to accumulation of reactive oxygen species (ROS), their subsequent damages and tissue deformities. Moreover, the diseases and disorders caused by OS include cardiovascular diseases [CVDs], chronic obstructive pulmonary disease, chronic kidney disease, neurodegenerative diseases and cancer. OS, induced by ROS, is neutralized by different enzymatic and non-enzymatic antioxidants and prevents cells, tissues and organs from damage. However, prolonged OS decreases the content of antioxidant status of cells by reducing the activities of reductants and antioxidative enzymes and gives rise to different pathological conditions. Therefore, the aim of the present review is to discuss the mechanism of ROS-induced OS signaling and their age-associated complications mediated through their toxic manifestations in order to devise effective preventive and curative natural therapeutic remedies.

## 1. Introduction

The foremost evolutionary outcome following various metabolic changes is the ability to bind energy in the form of adenosine triphosphate (ATP). Enzymes involved in various metabolic pathways produce energy through different biochemical reactions that prompt the final reduction in molecular oxygen (O_2_). Different processes of amphibolic metabolic pathways such as the tricarboxylic acid (TCA) cycle and the respiratory chain are linked to the inner mitochondrial membrane, which produces reactive oxygen species (ROS) and generates free radicals. The generation of free radicals is a basic and useful incident for the proper functioning, protection and survival of cells within physiological limits [1,2,3]. However, an imbalance in the generation and neutralization of ROS may lead to the accumulation of ROS intermediate products which are thought to be detrimental and may induce oxidative stress (OS) [1,4,5]. ROS are essential for maintenance of homeostatic environment for the cell survival, because they play a significant physiological role in defense mechanisms [6]. Therefore, ROS are important for the survival as well as adaptation, for instance, hydrogen peroxide and superoxide anion are substantial factors which are involved in the developmental signaling transduction in the pancreatic β-cells having ability to control insulin secretion [7,8,9]. However, the concentration of ROS may increase rapidly and reach toxic levels after calcium ion stimulation of the TCA cycle, an increase in the activity of respiratory chains, the transport of electrons and the activity of NADPH [10]. Furthermore, oxidative stress may be increased in the cell or tissue if there will be elevation in the rate of production and alleviation in the rate of neutralization of ROS [11]. Prolonged higher OS leads to various life-threatening pathological conditions such as aging, heart diseases, diabetes, autoimmune diseases, cancer, neurological disorders, etc. [12]. Elucidating the underlying mechanisms of OS-induced pathogenesis of numerous clinical conditions and aging will help in the development of therapeutic agents against such diseases and disorders for the overall well-being of humans.

### 1.1. Mechanism of ROS

Mitochondria are the primary endogenous source for ROS production due to their role in the ATP production via oxidative phosphorylation, during which the molecular O_2_ becomes reduced into H_2_O in electron transport chain (ETC). Mitochondrial superoxide production is an important source of ROS in cells. Seven major sites of superoxide production are recognized in mammalian mitochondria. In a descending order of maximum capacity, they are the ubiquinone binding sites in complex I (site IQ) and complex III (site IIIQo), glycerol 3-phosphate dehydrogenase (GPDH.), the flavin in complex I (site IF), the electron transferring flavor protein: Q oxidoreductase (ETFQOR) of fatty acid beta oxidation, and pyruvate and 2-oxoglutarate dehydrogenases. [13]. All these complexes release O^−^_2_ in the mitochondrial matrix except complex III site and GPDH. In the mitochondrial membrane, superoxide dismutase is present in three forms viz. manganese superoxide dismutase (Mn-SOD), copper superoxide dismutase and zinc superoxide dismutase. Manganese superoxide dismutase (Mn-SOD) converts O^−^_2_ into hydrogen peroxide (H_2_O_2_) and then aconitase enzyme converts H_2_O_2_ to a hydroxyl radical via Fenton reaction [14], while copper superoxide dismutase and zinc superoxide dismutase change O^−^_2_ into the inter membrane space [15].

**Figure 1:**
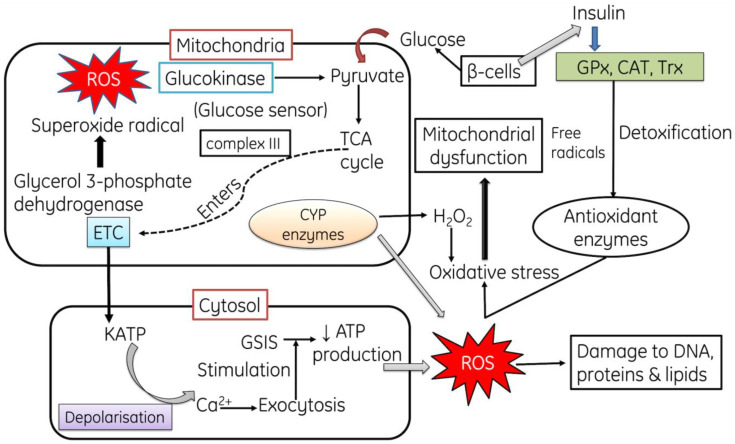
The mechanism for the production of ROS: Autoxidation with excess glucose alters the overall metabolic process and produces free radicals, inhibiting the antioxidant enzymes (GPX, CAT). ROS cause DNA damage. GPx: Glutathione peroxidase, CAT: Catalase, Trx: Thioredoxin, GSIS: glucose-stimulated insulin secretion.

Another route of ROS generation is mitochondrial cytochrome catalytic cycle, it comprises Cytochrome P450 enzyme consisting of wide spectrum of organic compounds, such as lipids, steroids, and xenobiotics. It produces various reactive byproducts such as superoxide radical and H_2_O_2_ [16,17]. Moreover, in mammals, a range of protein complexes also produce ROS, such as Nicotinamide adenine dinucleotide (NADH)-cytochrome b5 reductase (b5R) [18], dihydroorotate dehydrogenase (DHODH) [19,20], complex II: succinate dehydrogenase (SDH) [21,22], and monoamine oxidases (MAO) [23]. Several defense systems, in the form of antioxidants like GPXs, TRXPs, SODs, PRDXs, GSH, TRX2, GRX2, cyt c oxidase (complex IV), coenzyme Q, ascorbic acid, vitamin E and carotenes, protect the mitochondria from detrimental effects of ROS [23,24,25,26]. However, overproduction of ROS is associated with different pathologies in human beings such as mitochondrial dysfunction, cancer, inflammation, neurological and neurodegenerative diseases, diabetes, chronic renal diseases, aging and DNA damage [27,28,29,30]. Genomic DNA and/or mitochondrial DNA damage, mutations in somatic cells, instability in genome, oncogene activation, inhibition of tumor suppressor genes, changes in various pathways of metabolism and signaling with the immediate activation of compensatory mechanisms, all contribute to cellular damage [31,32] (Figure 1).

### 1.2. Mechanism of ROS-Mediated Toxicity

Cells always strive to maintain the level of ROS required for its normal functioning. However, excess production of ROS reduces the activity of antioxidant enzymatic defense system and also decreases the content of non-enzymatic proteins (GSH), hence affecting the overall antioxidative defense system and making it unable to eliminate the surplus free radicals. These excessive ROS are produced under hyperoxia and inflammatory conditions, and a low or impaired antioxidant defense system, and finally alter the homeostasis of the whole biological system. For instance, the oxidation of proteins and production of carbonyl and nitrotyrosine, i.e., nitrosylation might decrease the activity of enzymes and also reduces the production of growth factors that might result in cellular dysfunction [33]. One of the biomarkers for the assessment of ROS-mediated damage is lipid peroxidation (LPO), which indicates the extent of peroxidative damage on membranous phospholipids through the activation of sphingomyelinase activation and ceramide release and finally causes cell death [34]. The excessive generation of ROS causes oxidative deoxyribonucleic acid (DNA) damage. ROS can react with the nucleic acids by attacking the nitrogenous bases and the sugar phosphate backbone, thus inducing single- and double-stranded DNA breaks, which are also associated with premature aging [35].

### 1.3. ROS as Second Messengers

ROS can act as second messengers in some situations, and their optimum concentrations activate a variety of signal transduction pathways in the cell and facilitate the actions of growth factors like cytokines, and Ca^2+^ signaling. Furthermore, the role of ROS can be explained through the c-Jun N-terminal kinases (JNKs), a member of protein kinase family which is activated by ROS through the production of lipid peroxide intermediates and then phosphorylated. Following its phosphorylation, it releases two B-cell lymphoma 2-related proteins (BCL2) and sequestered in the cell [36,37]. After the release of these proteins, they directly activate theBCL2-associated X (Bax) proteins through cytoplasmic anchor.

Hydrogen peroxide (H_2_O_2_) is an essential metabolite involved in various redox metabolic reactions and cellular processes. It has been identified that H_2_O_2_ acts as a sensor, modulator and signaling molecule during redox metabolism, and also works as a second messenger along with hydrogen sulfide (H_2_S) and nitric oxide (NO) [38,39,40]. Following the activation of these second messengers, they activate a downstream protein cascade through specific oxidations, thus leading to the cellular metabolic response [41,42,43]. This metabolic response can influence proliferation, survival or death of the cell depending on which downstream pathways (homeostatic, pathological or protective) have been activated [44]. In cells, H_2_O_2_ is produced from various sources such as either a spontaneous or catalytic breakdown of superoxide anion, produced through the partial reduction of oxygen during aerobic respiration or through various oxidases, and is quickly converted into H_2_O_2_ by superoxide dismutase present in the mitochondria or cytosol or in the extracellular space [45,46,47] and regulatory machinery of the cell maintains the concentration in different sub-cellular compartments. 

In biological systems and in medicine, H_2_O_2_ is used as anti-infective agent, such as for the cleaning of wounds, due to its strong oxidative potential to kill microorganisms and cells [38,48]. Hydrogen peroxide contributes to the maintenance of homeostatic metabolism, because it is a key molecule in the Third Principle of the Redox Code “Redox sensing by activating/deactivating cyclic production associated with NAD and NADP system to support spatiotemporal organization of key processes” [49,50,51] of living organisms and contributes to the regulation of cellular metabolism. H_2_O_2_ diffuses through the cell membrane through aquaporin water channels (AQP) transducing the redox signal from the place where it is produced to a target site [52]. H_2_O_2_ activates various transcription factors in bacteria, lower eukaryotes and mammalian cells [53] and this stimulation finally leads to a metabolic response of the cell to the original stimuli [54]. 

### 1.4. ROS Induces Multifaceted Alterations

An imbalance between free radicals and oxidants gives rise a tense-full situation in the living system known as oxidative stress. Oxidative stress is a harmful process because it adversely affects the structure of cell membranes, lipids, proteins, lipoproteins and deoxyribonucleic acid (DNA) [55,56,57,58,59,60,61]. Likewise, the excessive production of hydroxyl radicals and peroxynitrile leads to peroxidation of lipids; hence, this damages the cellular membrane and lipoproteins. This in turn results in the formation of malondialdehyde (MDA) and conjugated diene compounds; both of these are known as cytotoxic and mutagenic compound. The rate of lipid peroxidation increases very rapidly and affects a huge quantity of lipidic molecules [62]. Due to peroxidative damage, proteins are also affected by OS, undergoing structural modifications that may indicate loss or an impairment of their respective enzymatic activity [59,63].

**Figure 2:**
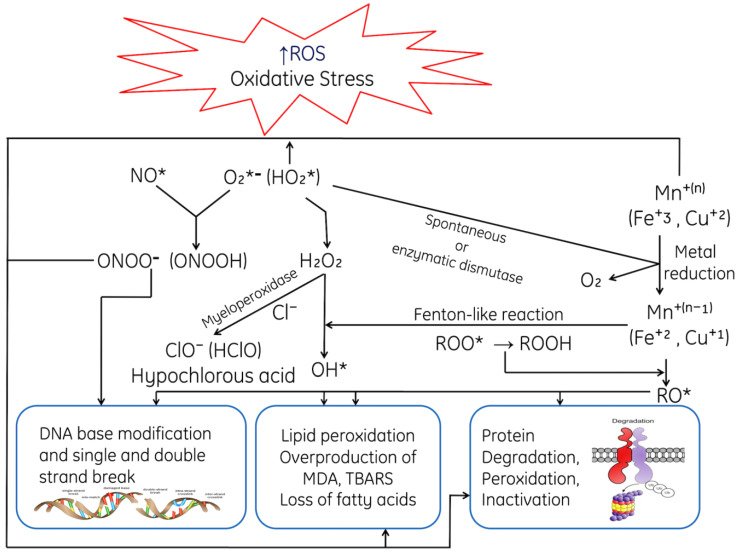
ROS-mediated toxic manifestations such as ROS-mediated DNA damage; peroxidative damage of membrane lipids, degradation of proteins caused ROS, Base modifications of Bases, where MDA: Malondialdehyde, TBARS: Thiobarbituric acid-reactive species, *, unpaired electron.

Prolonged OS causes lesions in DNA; the most common base affected is guanine bases in DNA and form 8-hydroxydeoxyguanosine (8-OHdG), which can bind to thymidine rather than cytosine. The level of 8-OHdG is generally considered to be a pernicious DNA lesion and is responsible for the mutagenesis [64]. Oxidative DNA damage can lead to the loss of epigenetic information, possibly due to the damage in CpG island methylation asset in promotor regions of a gene [65]. Moreover, it has been studied that 8-OHdG acts a tissue biomarker of oxidative stress. Therefore, considering all these consequences, it can infer that OS may induce various diseases (acute and chronic) speedup ageing processes of cells and also causes acute pathologies (trauma and stroke). Prolonged and higher OS leads to various life-threatening pathological conditions such as cardiovascular disease [66], respiratory diseases [67,68], rheumatoid arthritis [69], neurological disorders [70], liver and kidney diseases, reproductive diseases and diabetes [71,72]. Oxidative stress initiates various apoptotic signaling pathways due to increased production of ROS or reduction in the activities antioxidative enzymes, disruption of intracellular redox homeostasis and peroxidative damage in lipids, proteins or fragmentation of DNA. ROS-mediated toxic manifestations have been explained in Figure 2. 

### 1.5. Physiological Impacts of ROS

Reactive oxygen species (ROS) protect the body from pathogens such as microbes and foreign agents. At a physiological level, ROS destroy pathogens; hence, they act as an immune system to provide defense against foreign bodies [73], and are also used for the formation of cell structures such as protein complexes [74]. Cells may produce ROS exogenously and they are used for intracellular signaling and also for exciting redox-sensitive signaling pathways because ROS increase the production of cytoprotective regulatory proteins [1,75]. Therefore, ROS control pro-inflammatory signaling, pro-fibrotic signaling, the proliferation of cells, cell death and a variety of other biological processes without activating a necessity for macromolecular damage [76,77].

The redox state of sulfur switches comprises three central proteins complexes such as Cys/CySS, GSH/GSSG and thioredoxins (Trx) that maintains the equilibrium values of redox protein complexes in cellular organelles [78,79]. For the maintenance of the structure of proteins, trafficking of protein, enzyme regulation, signaling of cells and receptors, various transcription factors sulfur switches are used widely [80]. An imbalance in the ROS system in favor of surplus production of oxidants like hydroxyl ions might cause damage to the macromolecules, because the primary target of ROS including hydrogen peroxide (a mild oxidant) is the –SH group of the Cys residues of the protein [81,82]. However, thioredoxin and peroxiredoxin are able to reduce disulfide bonds and protein sulfenic acids and act as mediators in redox signaling [83].

An accumulation in the ROS levels inside the cells leads to Cys oxidation residues in intermembrane proteins such as kinases and phophatases due their contact with various stimuli, eventually affecting the signal transduction processes [84]. The oxidized species affect the growth factors, cytokines and hormones, or can induce the downregulatory effect to signaling pathways such as PKa, mitogen-activated protein kinase (MAPK) and phosphoinositide 3-kinase (PI3K) cascades [75]. Oxidants also inhibit the activity of phosphatases via the oxidation of the reactive cys residue and inhibit its catalytic activity [85].

## 2. Effect of ROS on Human Health

The imbalance between the production and the elimination of free radicals leads to OS inside the human body, which indicates that the level of oxidants overpowers the antioxidant system and then these oxidants negatively affect the various cellular structures, including membranes, proteins, lipids and DNA [1,86,87,88,89,90]. A well-known biomarker for the assessment of (OS) is lipid peroxidation (LPO). The rate of LPO increases rapidly during OS due to the peroxidative damage caused by free radicals on the polyunsaturated lipid membrane. Moreover, OS also cause conformational changes in proteins, thus decreasing their functional efficiency and alter their enzyme activities [89].

DNA damage and mutations induced by OS are associated with different human pathologies including cancer. OS induces lesions in DNA, which reveals that ROS-induced OS could lead to gene and chromosome mutations through DNA double-strand breaks (DSBs) [91,92]. It has been reported that OS-induced DNA damage leads to the increased production of mutagenic base8-oxo-2′-deoxyguanosine levels in a tissue which acts as a biomarker for OS [93].

**Figure 3:**
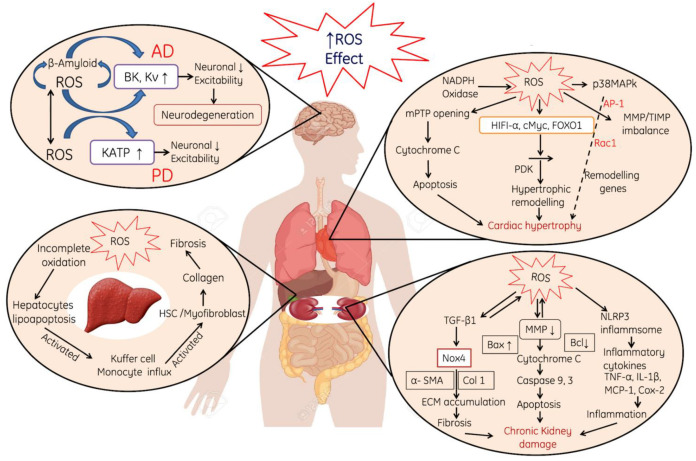
Effect of reactive oxygen species (ROS). ROS generation induces Alzheimer’s disease by accumulation of β-Amyloid proteins, activation of BK and Kv and finally decreases excitability property of neurons and neuro-degeneration of the heart, liver and kidneys, where KATP; ATP-sensitive potassium, NADPH oxidase: Nicotinamide dinucleotide Phosphate reduced, p38MAPK; p38 mitogen-activated protein kinases, MMP; Matrix metalloproteinases/tissue inhibitor of metalloproteinases, HIFA-α; Hypoxia-inducible factor 1-alpha, FOXO1; Forkhead box transcription factors, mPTP: mitochondrial permeability transition pore, PDK: Protein 3-phosphoinositide-dependent protein kinase, NLRP: Nucleotide-binding oligomerization domain, MCP: Monocyte Chemoattractant Protein, TNF-α: Tumor necrosis factor alpha, IL-1β: Interleukin-1 beta. Note: Upward arrows indicate increase and downward arrows indicate decrease.

ROS-induced OS causes alterations in different organ systems, which are explained as follows:

1. ROS-induced neurotoxicity causes modulation in the neurons such as permeabilization of the cellular membrane, a decrease in the excitability property of neuronal membrane and the activation of the KATP pump (Figure 3).
2. ROS-induced oxidative stress also leads to cardiac myopathy due to the mitochondrial damage, perforations in the mitochondria, the release of Cytochrome C and the initiation of apoptotic cascade (Figure 3).
3. Liver and kidneys are the primary targets for ROS attack due to their direct involvement in metabolic and filtration processes. In the liver, ROS induce damages to the hepatocyte membranes and leads to deuteriation, which in turn leads to the deposition of collagen in the hepatocyte and finally cause liver fibrosis and cirrhosis. In addition, the incomplete oxidation of biomolecules causes lipoapoptosis in hepatic cells and induces immune reactions in the liver (Figure 3). In the kidneys, ROS-induced oxidative stress mainly initiates the production of various pro-inflammatory cytokines, which initiates nephrotic inflammation and finally affects the renal functions (Figure 3).

### 2.1. Role of ROS in Mitochondrial Dysfunction and in Diabetes Mellitus

Diabetes is a multifaceted metabolic disorder characterized by an abnormal increase in blood glucose levels, which may or may be not associated with the reduced secretion of insulin. Usually, the pancreatic beta cells adjust their secretion of insulin according to the fluctuations in the concentration of glucose in blood, sensed by glucokinase, a glucose sensor. However, during diabetes mellitus, glucose levels increase above the normal range for a prolonged period of time. This chronic hyperglycemia has several deadly negative impacts upon the structure and functions of different organs, viz. inducing neuropathy, retinopathy, nephropathy, hepatic diseases and cardiovascular impediments. These conditions arise due to the generation of superoxide radicals (O_2_*), hydroxyl peroxide and hydroxyl radicals (OH*) through auto-oxidation during the hyperglycemic condition.

**Figure 4:**
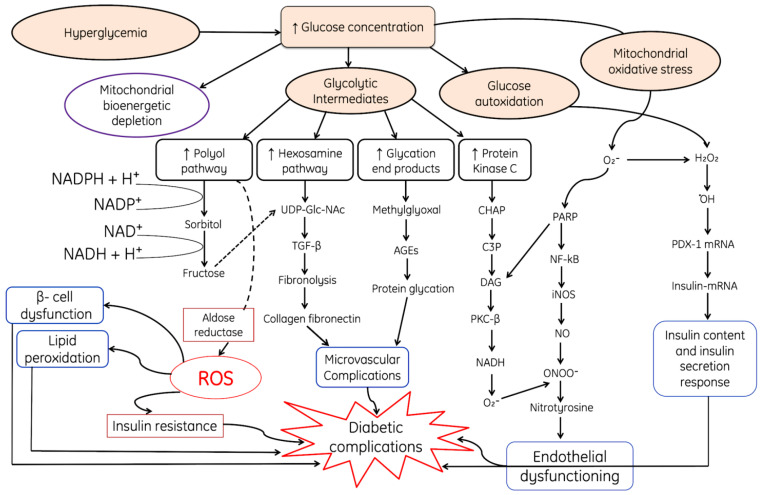
Hyperglycemic condition is one of the major sources of ROS and leads to modulation of various metabolic downregulatory pathways. Increased glucose results in auto-oxidation and production of H_2_O_2_, ·OH and activation of downregulatory pathways such as PDX-1 and insulin-(mRNA) and increases the secretion, where DAG: Diacyl glycerol, PKC-β: Protein kinase-beta, NF-κB: Nuclear factor kappa light chain enhancer of activated B cells, AGEs: Advanced glycated end products, UDP-Glac-NAc: Uridine diphosphate *N*-acetylglucosamine, TGF-β; Transforming growth factor beta, PDX1; pancreatic and duodenal homeobox-1, PARP: Poly (ADP-ribose) polymerase. Closure of K^+^-ATP-dependent channels results in membrane depolarization and activation of voltage-dependent calcium channels, leading to an increase in intracellular calcium concentration; this triggers pulsatile insulin secretion. Note: Upward arrows indicate increase and downward arrows indicate decrease.

Due to damage in pancreatic β-cells insulin secretion and signaling becomes hampered by mitochondrial toxicity or decreased oxygen supply to the mitochondria which hinders glucose-stimulated insulin secretion from beta cells [94,95,96]. The dysfunction of the mitochondria in these cells results in least formation of mitochondrial adenosine triphosphate which leads to the loss of glucose-stimulated insulin secretion [97,98]. Moreover, autoxidized glucose results in mitochondrial DNA damage [99] and the initiation of various ROS-and RNS-generating pathways, which finally leads to endothelial dysfunction (Figure 4). All these processes result in mitochondrial apoptosis and hamper ATP production [100,101,102,103,104]. Some studies have reported that insulin secretion might be hampered due to overexpression of uncoupling protein 2 (negative controller of insulin secretion) in the mitochondria of pancreatic β-cells, which leads to a decline in the generation of ATP and glucose-stimulated insulin secretion [105,106].

Hyperglycemic conditions during diabetes cause various complications such as hyperglycemia-induced autoxidation, resulting in the production of H_2_O_2_ and ·OH which affect the synthesis of insulin by damaging the insulin-mRNA, and finally decrease the content of insulin and also reduce the functional efficacy of the insulin response (Figure 4). Moreover, autoxidized glycolytic intermediates increase the production rate of glycation end product, initiate various pathways, such as the protein kinase C pathway, hexosamine pathway and the polyol pathway (Figure 4). The downregulatory end products of all these pathways ultimately lead to microvascular complications due to the accumulation of collagen fibronectin and protein glycation products (Figure 4).

### 2.2. Role of ROS in Obesity and Associated Comorbidities

Obesity is a chronic and complex disease involving an excessive amount of body fat and body mass index of 30 kg/m^2^ or higher, which increases the risk for other diseases and health-related problems, such as heart diseases [107], diabetes, high blood pressure and certain cancers. Due to serious health consequences, obesity is a leading cause of unnecessary deaths worldwide. Various factors generating OS during obesity include hyperglycemia, elevated tissue lipid levels, vitamin and mineral deficiencies, chronic inflammation, hyperleptinemia, increased muscle activity to carry excessive weight, endothelial dysfunction, impaired mitochondrial function and type of diet. All of these complications lead to excessive production of ROS due to the weakening of the antioxidant system. Excessive production of ROS is intricately associated with obesity and associated complications, especially insulin resistance and type 2 diabetes.

**Figure 5:**
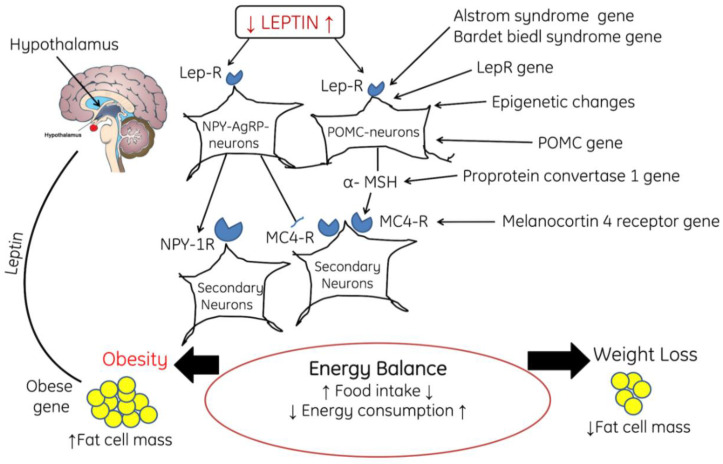
The diagram shows the role of feedback mechanism of leptin, POMC neurons, and NPY-AgRP neurons in the regulation of satiety and starving, where POMC: proopiomelanocortin, NPY/AgRP: neuropeptide Y/Agouti-related protein, Lep-R: Leptin receptor, α-MSH: Alpha melanin-stimulating hormone, MC4-R: Melanocortin receptor-4. Note: Upward arrows indicate increase and downward arrows indicate decrease.

Obesity-associated complications alter the signaling cascade in the body. One of the common pathways which is significantly affected during obesity is leptin signaling pathway. Leptin, a hormone having the ability to reduce the intake of food and body weight, was initially considered for use in the treatment of obesity. However, it has been reported that during obesity, the levels of circulatory leptin increase. It crosses the blood–brain-barrier (BBB) through a specific and saturable transporter. Therefore, at the neuronal level leptin indicates the degree of obesity of an organism. It has been reported that with the increase in adiposity, the serum leptin level also increases, which in turn leads to the development of resistance at the level of the BBB transporter [108,109]. This indicates that a smaller amount of leptin reaches the brain, thus leading to the activation of the signaling pathway for weight regulation. In the brain, leptin binds with leptin NPY-AgRP-neurons, which activate the NPY-1R and inhibit MC4-R on secondary neurons, finally leading to an energy imbalance and energy consumption and increasing the accumulation of fat cell mass (Figure 5).

### 2.3. Role of ROS in Cardiac Hypertrophy

**Figure 6:**
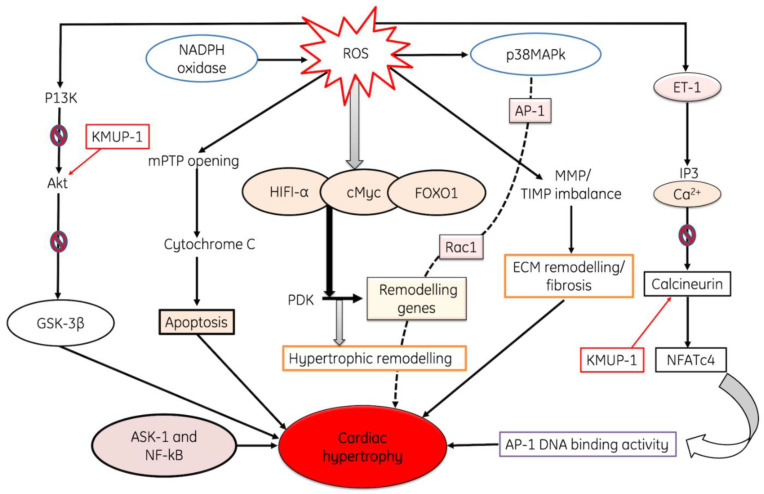
The diagram shows ROS-induced cardiac hypertrophy through the up-regulation of various pathways. ROS induces the activation of endotjelin-1 protein which in turn causes break down of PIP3 into IP3 and DAG, both of these activates the calcium channels and releases calcium from endoplasmic reticulum. Liberation of Ca from ER inhibits the activation of Calcineurin, also mediated by KMUP-1, and finally causes cardiac hypertrophy through the activation of AP-1DNA binding activity of NFATc4. Moreover, ROS activates HIFI-1, FOXO-α and Myc pathways and leads to the activation of hypertrophic remodeling. ROS also induce cardiac hypertrophy through the opening of mPTP channel, release of cytochrome C and induction of apoptosis of myocardial muscles. Additionally, ROS induces inhibition of P13K, Akt and GSK-3β. All these pathways induce cardiac hypertrophy, where ET-1: Endothelin-1, mPTP: Mitochondrial Permeability Transition Pore, ASK-1: Apoptosis signal-regulating kinase-1, MMP. TIMP: Matrix metalloproteinase/Tissue inhibitors of metalloproteinases, AP-1: Activator protein 1, NF-κβ: Nuclear factor-κ Beta, KATP; ATP-sensitive potassium, NADPH oxidase: Nicotinamide dinucleotide phosphate reduced, p38MAPK; p38 mitogen-activated protein kinases, HIFA-α; Hypoxia-inducible factor 1-alpha, FOXO1; Fork-head box transcription factors, PDK: Protein 3-phosphoinositide-dependent protein kinase, NLRP: Nucleotide-binding oligomerization domain, MCP: Monocyte Chemo-attractant Protein, TNF-α:Tumor necrosis factor alpha, IL-1β: Interleukin-1 beta.

Cardiac hypertrophy (CH) is an adaptive response of the heart to pressure overload. It is a general pathological characteristic in the natural course of some major cardiovascular diseases, such as hypertension and myocardial infarction. Cardiac hypertrophy is strongly associated with a higher risk for heart failure and sudden cardiac death. OS is considered as one of the important contributing factors in the development of cardiac hypertrophy. Different pathways are involved in the development of hypertrophy like Angiotensin-II (Ang II)-induced cardiac hypertrophy which is induced by an extracellular signal. ROS also activate a broad variety of hypertrophy signaling kinases and transcription factors, such as MAP kinase, NF-κB, etc. In addition to the profound alteration of cellular function, ROS modulate the extracellular matrix function, evidenced by increased interstitial and perivascular fibrosis. AngII stimulates the expression of the hypertrophic factor, beta-myosin heavy chain genes in myocytes, and is mediated via ROS-dependent activation of the extracellular-*signal*-regulated *kinase signaling cascades* [110]. A study was conducted on guinea pigs to evaluate progressive left ventricular hypertrophy, and it has been found that the expression of p22^phox^, p67^phox^, p47^phox^, Nox2 and Nox activity was upregulated during the progression of cardiac hypertrophy to failure [111]. Additionally, Nox can be stimulated by angiotensin-II to generate ROS, causing the activation of p38MAP kinase following AP-1 activation, while it has also been confirmed that the activation of Rac1 is a critical step in hypertrophy progression [112]. Moreover, NADPH oxidase also increases the production of ROS, which further accelerates these pathways. ET-1 causes hydrolysis of phosphatidylinositol 4,5-bisphosphate (PIP2) into IP3 and DAG, both these secondary messengers activate calcium channels in the ER and releases Ca from it. Both Ca and KMUP-1 activate the calcineurin, and it in turn actives NFATc4; finally, it increases AP-1 DNA binding activity and initiates cardiac hypertrophy. ROS activation also activates HIF1-α, cMyc, FOXO1 and PDK, and finally causes the activation of hypertrophic remodeling [113]. ROS also causes damage to the mitochondria, and cytochrome C is released from the mitochondria, which finally leads to apoptosis [114,115,116]. All of these processes collectively result in cardiac hypertrophy (Figure 6)

## 3. Oxidative Stress and Neurodegenerative Diseases

### 3.1. Alzheimer’s Disease

Neurodegenerative diseases impact not only the life quality of aging populations, but also their life spans. All forms of neurodegenerative diseases have a wide range of impact on the elderly. The major threat of these brain diseases includes progressive loss of memory, Alzheimer’s disease (AD), impairments in the movement, Parkinson’s disease (PD) and Huntington’s disease (HD). Alzheimer’s disease (AD) is one of the utmost neuro-degenerative disorders that affect the elderly. During AD accumulation of amyloid beta protein, neuronal or synaptic loss and brain atrophy in particular areas of the brain occurs. AD affects the neocortex as well as the hippocampus; brain plaques and tangles are the key characteristics of AD. An imbalance between the oxidant-antioxidant systems could determine the degree of cellular damage. Oxidative stress and mitochondrial dysfunction are key hallmark features of neurodegeneration [117], and plays an important role in the progression of neuronal damage during AD [118]. This ROS-mediated neuronal damage occurs due to the disruption of redox reaction, i.e., decrease in the activities of antioxidant enzymes such as superoxide dismutase and catalase activities, and a decrease in the level of antioxidants (ascorbic acid and tocopherol) favors the surplus ROS production along with increased accumulation of Aβ in AD patients [119]. Aβ plaques are deposited in the extracellular region of the endoplasmic reticulum and Golgi apparatus. This increase in the deposition of Aβ plaques during initial stages of AD also increases oxidative stress and finally leads to mitochondrial dysfunction [120,121]. Increased expression of amyloid mutant amyloid precursor protein and presenilin-1 increases the levels of hydrogen peroxide (H_2_O_2_) and lipid peroxidation [119,122]. It also promotes the phosphorylation of tau protein in AD patients [119]. It has been studied that removal of cytoplasmic/zinc superoxide dismutase (SOD) drives the β-Amyloid protein oligomerization and memory Loss in a mouse model of Alzheimer’s disease [123,124]. Previous studies reported that overexpression of tau protein increases susceptibility of oxidative stress and reduction of peroxisomes [125]. Besides this, overexpressed mutant tau proteins (P301S and P301L) decrease the activity of nicotinamide adenine dinucleotide hydrogen-oxidoreductase, leading to mitochondrial dysfunction. Both factors are responsible for the ROS production [126,127,128]. It has been reported this mitochondrial dysfunction inhibits the efficient generation of adenosine triphosphate (ATP) [119,129]. Moreover, mitochondrial dysfunction in neuronal cells also downregulates the expression of electron transport chain (ETC) genes responsible for the activation of pyruvate, cytochrome oxidase and α-ketoglutarate dehydrogenase [130,131].

**Figure 7:**
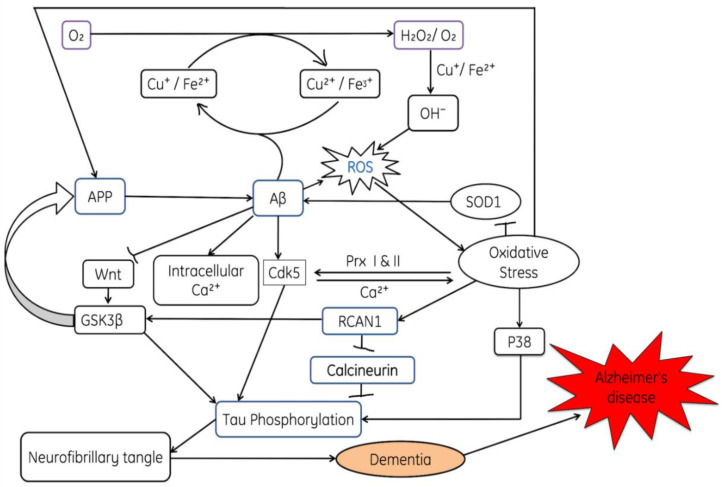
The diagram shows the involvement of reactive oxygen species and amyloid β in Alzheimer’s disease, where Aβ; amyloid plaques, NFT: neurofibrillary tangles; AD: Alzheimer’s disease, SOD: superoxide dismutase, CAT: Catalase, APP: Amyloid β (Aβ) precursor protein, GSK3β: Glycogen synthase kinase-3, Wnt-1: Wingless and Int-1, Cdk5: Cyclin-dependent kinase-5, PRx: peroxiredoxin, RCAN1: regulator of calcineurin 1.

ROS-induced inactivation of antioxidant enzyme activities such as superoxide dismutase (SOD), activation of p38 and phosphorylation of tau protein causes an accumulation of neurofibrillary tangles, which are represented in Figure 7. Moreover, the role of ROS in activation of cdk5, the release of intracellular Ca^2+^ and the inhibition of the wnt pathway are also explained in Figure 7 Due to the inactivation of the wnt pathway, GSK3β becomes activated and then favors the phosphorylation of tau proteins.

### 3.2. Parkinson’s Disease

Parkinson’s disease is a progressive nervous system disorder that affects movement. Common symptoms include tremors, slowness of movement, stiff muscles, unsteady walk and balance and coordination problems. Symptoms start developing slowly, occasionally starting with a barely noticeable tremor in just one hand. Tremors are common, but the disorder also commonly causes stiffness or slowing of movement. Various sources and mechanism are involved in the generation of ROS viz metabolism of dopamine itself, mitochondrial dysfunction, iron, neuro-inflammatory cells, calcium and aging. The gene product involved in the development of PD includes DJ-1, PINK1, parkin, alpha-synuclein and LRRK2, and also impacts mitochondrial function in complex ways, leading to the aggravation of ROS generation and vulnerability to OS. Moreover, OS influences the cellular homeostatic processes including ubiquitin-proteasome system and mitophagy. Interaction among all these mechanisms contribute to neurodegeneration in PD due to OS, which damages important cellular pathogenetic proteins that causes surplus generation of ROS. Parkinson’s disease (PD) is distinguished by discriminating neuronal loss of dopaminergic neurons in the substantia nigra pars compacta (SNc) and reduced dopaminergic level in the nigrostriatal dopaminergic pathway in the brain [132,133]. It is thought that OS-induced pathophysiological condition is the fundamental cause of Parkinson’s disease (PD) [133,134]. Recent studies reported that decreased activity of respiratory complex I in substatia nigra pars compacta of patients suffering from Parkinson’s disease may cause excessive ROS generation and induce cellular loss of dopaminergic neurons [133,134,135]. 

The inhibitors such as 1-methyl-4-phenyl 1,2,3,4-tetrahydrpyridine (MPTP) and its metablites, 1-methyl4-phenylpyridinium (MPP+) of Complex I may lead to the cytotoxic effects on the dopaminergic neurons, resulting in clinical parkinsonian phenotype, and induce nigral degeneration with cytoplasmic α-synuclein [136]. Genetic mutations in proteins such as α-synuclein, parkin, and phosphatase and tensin homolog induce putative kinase that were associated with the ancestral forms of Parkinson’s disease [133]. The functions of mitochondria have been affected when these genes are mutated and increases ROS-induced oxidative stress [133]. Some studies evidenced that point mutations and deletion in the mitochondrial DNA might cause a defect(s) in complex I or mitochondrial dysfunction in Parkinson’s disease [137] and encode Complex I subunits in patients suffering from PD [137,138,139]

**Figure 8:**
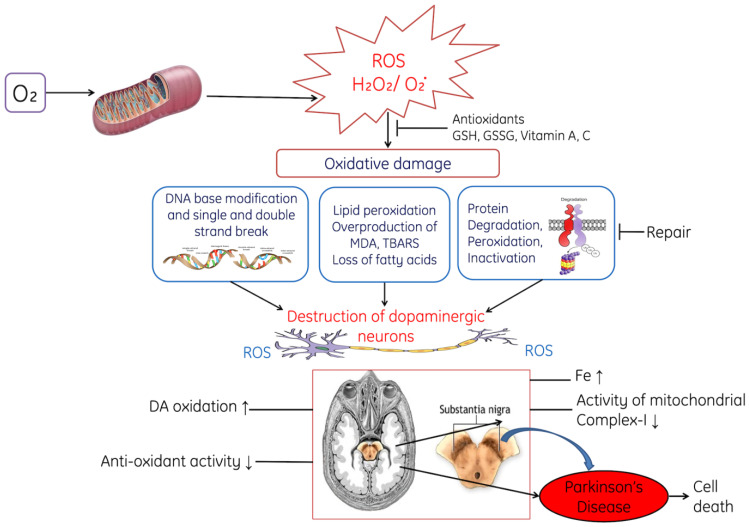
The diagram shows the role of ROS in Parkinson’s disease through the of dopaminergic neurons. ROS exerts oxidative stress on DNA leads to modification in base and causes fragmentation in DNA. Peroxidative damage in lipids, loss of fatty acids, changes in plasma membrane and degradation of proteins. All these changes damage the dopaminergic neurons, where H_2_O_2_: Hydrogen peroxide; ROS: Reactive oxygen species, GSH: Reduced glutathione, GSSG: Oxidized glutathione.

Moreover, ROS-induced destruction of dopaminergic neurons is shown in Figure 8. ROS-induced oxidative stress decreases the level of reduced glutathione (GSH) in the substantia nigra pars compacta of PD [140]. Increased oxidative stress accelerates the rate of lipid peroxidation, due to the degradation of membranous polyunsaturated lipids and degradation of protein, and ROS also induced the fragmentation of DNA. All these processes ultimately lead to the damage of dopaminergic neurons and results in lack of coordination among the body parts. Additionally, experimental studies conducted on animal models have reported that the levels of Fe increase in the substantia nigra pars compacta, which might arise due to dysfunctional transport of Fe to the mitochondria during Parkinson’s disease [141,142]. 

## 4. Oxidative Stress and Chronic Kidney Diseases

Kidney diseases have become a serious health concern throughout the world. Late diagnosis is one of the major concerns. The causes for increasing levels of kidney disease include various factors such as an increased rate of diabetes, high blood pressure, prescription of allopathic medicines, xenobiotics, etc. These factors lead to the formation of ROS and cause oxidative stress in the renal system. Oxidative stress is one of the major progressive threatening factors which is responsible for the increasing prevalence of chronic kidney diseases (CKD) or end-stage renal diseases (ESRD), and may even lead to death [143,144]. 

It has been reported that during initial stages of CKD, the intensity of oxidative stress becomes accelerated [145], which autonomously results in the development of ESRD [146]. This progression from CKD to ESRD has been observed in patients undergoing Hemodialysis (HD) [146,147]. Patients with ESRD on peritoneal dialysis (PD) have amplified OS in comparison to non-dialysis uremic patients; however, it is less when compared with patients undergoing HD [148,149]. In the kidneys, OS is responsible for progressive kidney damage, such as lesions to the glomeruli, renal ischemia, apoptosis and cell death, intensifying the unadorned ongoing inflammatory processes. ROS-induced OS modulates various signaling pathways, enzymes and gene expressions in the renal system.

NADPH oxidase (Nox) leads to the production of reactive ROS and contributes significantly to the progression of renal diseases. Nox encompasses seven members (Nox 1–5, DUOX1, DUOX2), and all these isoforms differ from one another on the basis of their respective functions: Nox1, Nox3 and Nox5 produce superoxide (O_2_*^−^), whereas DUOX1, DUOX2 and Nox4 mainly produce hydrogen peroxide (H_2_O_2_) [150]. For the activation of Nox1, various membrane subunits, such as p22phox, cytosolic subunits p47phox and p67phox, are required. Nox4 is the principal isoform of NADPH in the kidneys. Nox4 plays a significant role in redox processes involved in diabetic nephropathy, acute kidney injury, obstructive nephropathy, hypertensive nephropathy, renal cell carcinoma and other renal diseases by activating many signaling pathways [151]. Even though Nox4 is found in different cells viz epithelial cells, podocytes, mesangial cells, endothelial cells and fibroblasts, it can induce cell apoptosis, inflammation and fibrogenesis [152].

As mentioned in the previous section of this paper, amongst the DNA bases, guanine is most susceptible to oxidative stress and 8-OH-dG (8-hydroxy-20–deoxy-guanosine) is one of the abundant oxidative products of nucleic acids [153]. This results in oxidative damage to nucleic acids and amplifies the signal for the commencement of consequent tumors [154]. Other indicators of OS include oxidized low-density lipoprotein, advanced glycation end products (AGEs), 8-hydroxyde-oxyguanosine and malondialdehyde (MDA), which increase significantly in blood and other tissues in patients suffering from chronic kidney disease [155]. AGEs interact with cells through a receptor system for advanced glycation end products and the receptor for advanced glycation end-products (RAGE) [156], which in turn stimulates the mitogen-activated protein kinase transduction pathway. Such stimulation is responsible for the translocation of the p65 subunit of nuclear factor kappa B from the nucleus into the cytoplasm of the cell; this further stimulates second messengers and results in a subsequent increase in pro-inflammatory enzymes, cytokines and adhesion molecules [157,158,159]

### ROS-Mediated Cardio Vascular and Nephropathy

**Figure 9:**
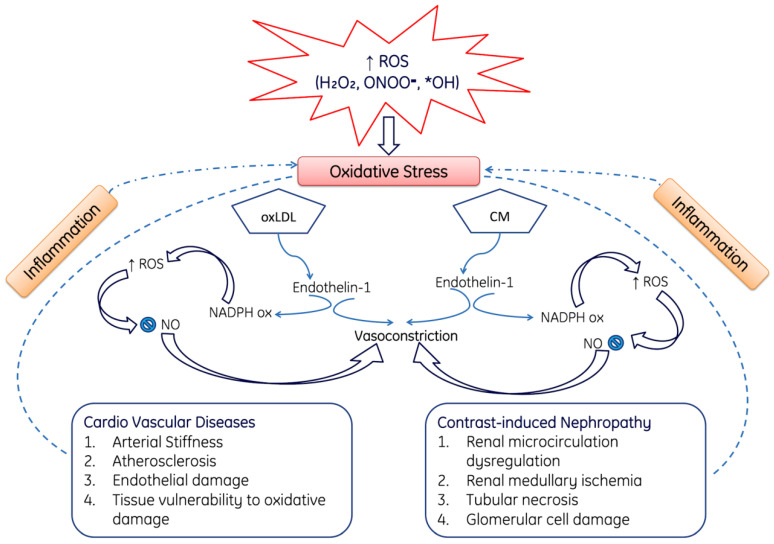
This diagram represents the role of ROS in the pathogenesis of cardiovascular cardiovascular diseases and contrast-induced nephropathy. Over produced reactive oxygen species such as H_2_O_2_, ONOO^−^, *OH induces oxidative damage on lipids (OxLDL) and CM and leads in the production of Endothelin-1. Endothelin-1along with ROS-mediated inflammation causes arterial stiffness, atherosclerosis, endothelial damage, tissue injury. Among the renal complications it causes renal microcirculation dysfunction, renal medullary ischemia, tubular necrosis and damages glomerular injury. Were, OxLDL: Oxidized low-density lipoproteins, CM: Cell membrane, NO: Nitric oxide, NADPH ox: nicotinamide adenine dinucleotide phosphate oxidase. Note: Upward arrows indicate increase and downward arrows indicate decrease.

Cardiovascular disease is one of the major causes of death in the entire world, not only in the common population but also in persons suffering from chronic kidney disease (CKD). Therefore, it may be proposed that cardiovascular diseases and chronic kidney diseases are interrelated with one another. Moreover, the number of patients with cardiovascular diseases and chronic kidney disease cause increased morbidity and mortality among the all-renal dysfunction spectrum. During cardiovascular diseases and nephropathy, the overproduction of ROS induces stress and leads to the oxidation of LDL (oxLDL) and CM; both of these causes affect Endothelin-1, which in turn activates NADPH oxidase (Nox) and NO and finally leads to vasoconstriction. Moreover, ROS production can also directly cause nephropathy, including renal microcirculation dysfunction, renal medullary ischemia, tubular necrosis and glomerular cell damage [160,161,162] (Figure 9). Additionally, Figure 9 demonstrates how elevated OS causes contrast-induced nephropathy characterized by improper renal microcirculation, tubular necrosis and glomerular damage. Figure 9 also shows how oxidative stress induces the oxidation of lipids, which causes arterial stiffness, endothelial damage and atherosclerosis. 

## 5. ROS in Aging and Age-Related Diseases

Reactive oxygen and nitrogen species (RONS) are generated through various endogenous and exogenous processes, and their undesirable effects are neutralized by antioxidant defenses. Due to the imbalance between RONS generation and antioxidant defenses results in the induction of OS. Aging is a process during which tissues and organs lose their functions or do not function efficiently. The OS theory regarding aging states those age-associated functional losses occur due to the deposition of RONS-induced damages. Simultaneously, OS is associated with various age-related circumstances (i.e., cardiovascular diseases [CVDs], chronic obstructive pulmonary disease, chronic kidney disease, neurodegenerative diseases and cancer), including sarcopenia and frailty [163,164,165]. OS-induced aging and associated disorders cause deterioration in soft tissues and disrupt the homeostasis [166,167].

**Figure 10:**
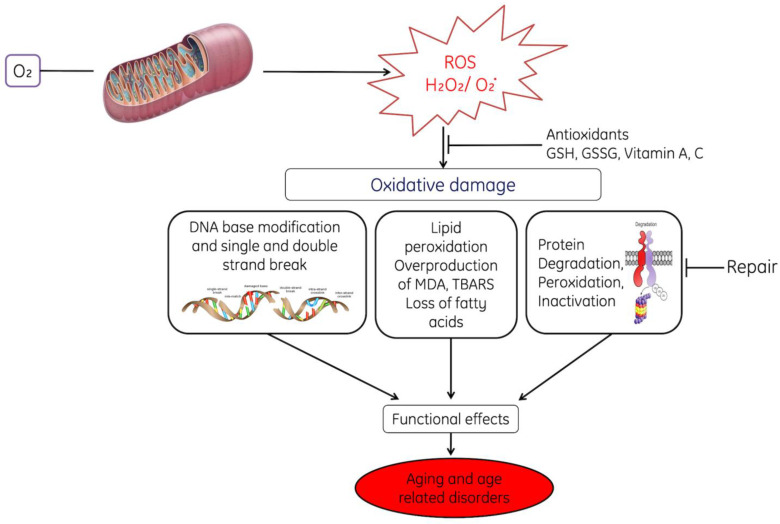
This diagram represents the role of reactive oxygen species (ROS) in cellular ageing. ROS-induced oxidative stress-induced damage in DNA (base alteration, fragmentation), peroxidative damage in lipids, protein degradation and inhibition of repair. All these abnormal changes in the cells decrease their working efficiency and causing their ageing. Were, GSH; Reduced glutathione, GSSG: Oxidized glutathione. MDA, TBARS. Note: Dot (.) designates free radical.

Moreover, OS induces abnormal signaling in mitochondria [168] which leads to changes in mitochondrial homeostasis such as modulation in the signaling of ROS and causes age-dependent cellular damage [169,170,171]. Progressive generation of ROS decreases the activity of scavenger enzymes and favors situation towards a pro-oxidant status which causes aging of cells [172,173,174]. It has been reported that depletion of mitochondrial superoxide dismutase and catalase causes elevation in OS in mice, which lead to premature aging and age-associated pathogenic conditions [175]. This increased oxidative-stress-induced mitochondrial dysfunction leads to aging of cells by reducing the life span of cells due to functional inefficiency of enzymes, proteins and other biomolecules [175,176,177,178,179] and accelerates the peroxidative damage to membrane lipids, decreases fatty acids and degrades the proteins (Figure 10).

ROS induce faults in replication and impair the mitochondrial DNA repair system, accumulation of aged protein and lipids and decrease the replicative capability of DNA [180,181,182,183,184,185] (Figure 10).

### Mitochondria-Associated Membranes Involvement with Aging

Sites of close contact between mitochondria and the endoplasmic reticulum (ER) are known as mitochondria-associated membranes (MAM) or mitochondria–ER contacts (MERCs). They contribute significantly to both cell physiology and pathology. Aging is a composite process associated with the steady weakening of cells, tissues and the entire function of an organism’s body with age. Aging influences the individual function of organelles, including mitochondria and the endoplasmic reticulum (ER), and hence affects their contact sites. At the cellular level, aging was associated with oxidative stress, accumulation of DNA modifications, impaired proteostasis and inefficient organelle turnover [186]. 

The endoplasmic reticulum (ER) and mitochondria are the chief intracellular organelles that are dynamically connected through temporally form of contact, called as mitochondria-associated membranes (MAMs). These contact sites are recognized due to their unique molecular composition, which is specifically restricted to the surrounding of the interacting membrane fragments. These molecular assemblies construct links in such a way that they develop a unique local environment, which could increase the transfer of cargo or signals among the different organelles. Previous studies have reported that MAMs form a physical platform which enables communication between the ER and mitochondria, which is associated with lipid biosynthesis. Moreover, the proteome of the MAMs fraction remains on target during the various age-associated disorders including Alzheimer’s disease [187,188], amyotrophic lateral sclerosis [189,190] and type 2 diabetes mellitus [191,192], as well as in obesity [193], GM1-gangliosidosis [194] and viral infections through human cytomegalovirus or hepatitis C virus [195,196].

Aging is considered one of the most important factors responsible for causing elevated ER stress and mitochondrial dysfunction, as well as the diminution of ER stress. Various studies have also reported that ER–mitochondrial contact plays important role in the biogenesis of mitochondrial-derived compartments [197,198,199], and these compartments might play vital roles in cellular adaptation to environmental stress conditions [199]. Aging is an important factor during which stress level increases in ER, which in turn leads to the mitochondrial dysfunction [200]. Moreover, various studies have also suggested that there is a correlation between spatial reorganization and elevated ATP levels, oxygen consumption, reductive power and elevated mitochondria Ca^2+^ uptake [201,202]. However, uncoupling of the organelles or blocking the Ca^2+^ transfer decreased the metabolic response, interpreting cells and makes them more prone to ER stress [201,203]. Subsequently, ER stress induces premature elevation in mitochondrial metabolism that relies mainly upon organelle coupling and the transfer of Ca^2+^ [201,202,203,204,205,206].

## 6. The Role of ROS in the Induction of DNA Damage

### 6.1. Role of ROS in Mediating Genotoxin-Induced Damage

Cells are exposed to endogenous as well as exogenous sources of ROS. Surplus level of ROS impairs physiological function through the cellular damage of DNA, proteins, lipids and other macromolecules. ROS-mediated harmful effects contribute to the progression of deteriorating conditions in human beings including neurodegenerative disorders, cardiac dysfunction, cancer, aging, etc. ROS induce deleterious effect on cells, and also act as stress-induced signaling molecules in cells. It has been reported that DNA damage is due to the increased production of intracellular ROS, and there are various sources which damage the DNA by ROS, such as ionizing radiations, synthetic chemicals, pesticides, etc. 

**Figure 11:**
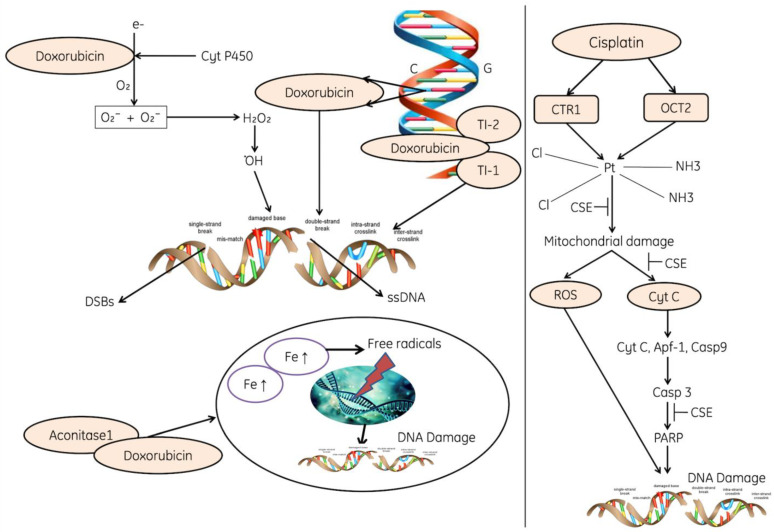
The diagram reflecting the role of cisplatin and Doxorubicin in the initiation of oxidative stress and molecular changes. Cisplatin activates the CTR1 and OCT2, damage in mitochondria (perforations), release of cytochrome, apoptosis and fragmentation in DNA. Doxorubicin induces the production of superoxides and peroxide production; ROS inhibit the enzymatic activities. Doxorubicin directly binds to the aconitase enzymes, enters into the nucleus and leads the ROS-induced DNA damage, where CRT1: The copper transporter CTR1 regulates1, OCT2; Organic cation transporter 2, PARP: poly (ADP-ribose) polymerase, APf-1: Apoptotic protease factor 1, Cyt C: Cytochrome C, Casp3, 9: Caspase3 and 9, and DSBs: Double-stranded breaks.NH_3_, Cl, CSE. Note: Dot (.) designates free radical.

Genotoxicity caused due to chemotherapeutics such as doxorubicin and cisplatin elevates the level of ROS, inhibits the antioxidant system such as antioxidants (GSH, and ascorbic acid), enzymes responsible for the regeneration of antioxidants, SOD, CAT and peroxidases [207,208]. The ROS causes perforation in the mitochondrial membrane and cytochrome C gets released and initiates the apoptosis. Moreover, cytochrome C, AfAP and Caspase 9 activate Caspase 3, which leads to the activation of PARP and finally causes DNA fragmentation (Figure 11). 

The other forms of DNA are also damaged due to the direct induction of ROS, these can oxidize nucleoside bases such as the formation of 8-oxo guanine, which can cause transversion of G-T or G-A if unrepaired [209]. The base excision repair pathway is used to recognize and repair oxidized bases, but when they are present simultaneously on opposite strands, attempted base excision repair can cause the formation of double-strand breaks [210]. The accumulation of ROS also induce lesion in the mitochondrial DNA, DNA strand breaks and deprivation of mitochondrial DNA (Figure 11).

### 6.2. Role of ROS-NO in DNA Damage by Oncogenic Replication Stress 

Cancer is caused due to alterations in critical regulatory genes that control cell proliferation, differentiation and survival. Studies have shown that specific genes called oncogenes are able to induce cellular transformation, thereby providing the first insights into the molecular basis of cancer.

DNA damage means physical or chemical changes to DNA in cells, which influence the interpretation and transmission of genetic information. There are various cancer-causing agents which are broadly divided into exogenous and endogenous; both of these include chemicals, radiations, free radicals and topological changes, and each of these agents induce distinct forms of damage [211]. Cells have evolved complex processes to deal with genomic damage. Depending on the nature of cuts in DNA, particular pathways are activated to facilitate recognition of the impaired regions and their repair [212,213]. A specific dangerous lesion is the DNA double-strand break (DSB), which may be mutagenic due to chromosomal rearrangement. To reverse the DNA damage, cells produce a network of events, known as the DNA damage response (DDR). This response includes recognition of damaged DNA, activation of checkpoints, cell cycle arrest and, ultimately, the outcome for repair, apoptosis and immune clearance [214,215]. The molecular components of the DSB-induced DDR have been studied broadly and broadly divided into three major groups viz. sensors which helps in the identification of damage. The transducer helps in coordinating the signaling and effectors, which ultimately facilitate the outcome [216,217,218].

ROS are well identified as mediators of DNA damage. Ionizing radiations (IR) induce double-stranded breaks by high-energy damage to the sugar backbone of DNA, and also through free radicals generated in cells—mostly •OH from water [22]. Chemotherapeutics including doxorubicin and cisplatin enhances the level of ROS and play an important role in their genotoxic effect [23,24]. It has been reported that ROS directly induce DNA damage by oxidizing nucleoside bases (e.g., the formation of 8-oxo guanine) [25] and convert G-T or G-A if unrepaired. The BER pathway identifies and repaired these oxidized bases [26]. Moreover, the accumulated ROS also induces lesions in mitochondrial DNA, breaks DNA strands and also causes degeneration of mitochondrial DNA [27].

## 7. Conclusions

Reactive oxygen species (ROS) are generated through different endogenous and exogenous processes. Oxidative stress is due to an imbalance between the ROS production and antioxidants defense and is progressive with ageing. Oxidative stress is also associated with various chronic diseases and damages such as diabetes, neuro-degenerative diseases, obesity, cardiac hypertrophy, hepatorenal damage, reproductive dysfunction, DNA damage, mitochondrial dysfunction and finally aging of cells. Biomarkers of OS might be useful as diagnostic tool or therapeutic target. Further investigations are needed to evaluate the efficacy of antioxidant supplements and natural polyphenolics from plant parts to combat the pathologies caused by oxidative stress. 

## 8. Future Perspective

Considering ROS-induced oxidative damages and diverse pathologies on different organ systems, it can be seen that oxidative stress leads to the evolution of numerous and diverse pathologies in the body. Further studies are required to elucidate the mechanisms through which oxidative-stress-related signaling can contribute to the alteration of different organs in order to devise effective preventive and curative therapeutic strategies. Various reports have revealed that the use of antioxidants can treat many diseases; the development of antioxidant therapies also represents a promising avenue for ROS pathology treatment. Antioxidant therapy, such as the use of polyphenols, flavonoids, antioxidant supplements and other nutritional compounds, together with moderate aerobic exercise, may reverse the damage induced by oxidative stress. Therefore, the knowledge of a particular antioxidant’s oxidative pathway associated with ROS could allow both the identification of disease markers and the development of preventive and curative therapeutic strategies. 

## References

[1] Dröge W. (2002). Free radicals in the physiological control of cell function. Physiol. Rev..

[2] Merry T.L., Ristow M. (2016). Mitohormesis in exercise training. Free Radic. Biol. Med..

[3] Phaniendra A., Jestadi D.B., Periyasamy L. (2015). Free radicals: Properties, sources, targets, and their implication in various diseases. Indian J. Clin. Biochem..

[4] Keane K.N., Cruzat V.F., Carlessi R., de Bittencourt P.I.H., Newsholme P. (2015). Molecular events linking oxidative stress and inflammation to insulin resistance and β-cell dysfunction. Oxid. Med. Cell. Longev..

[5] Araki E., Nishikawa T. (2010). Oxidative stress: A cause and therapeutic target of diabetic complications. J. Diabetes Investig..

[6] Finkel T., Holbrook N.J. (2000). Oxidative stress and aging: Catalase is a longevity determinant enzyme. Nature.

[7] Newsholme P., Cruzat V., Arfuso F., Keane K. (2014). Nutrient regulation of insulin secretion and action. J. Endocrinol..

[8] Evans J.L., Goldfine I.D., Maddux B.A., Grodsky G.M. (2003). Are oxidative stress-activated signaling pathways mediators of insulin resistance and β-cell dysfunction?. Diabetes.

[9] Newsholme P., Haber E.P., Hirabara S.M., Rebelato E.L.O., Procópio J., Morgan D., Oliveira-Emilio H.C., Carpinelli A.R., Curi R. (2007). Diabetes associated cell stress and dysfunction: Role of mitochondrial and non-mitochondrial ROS production and activity. J. Physiol..

[10] Newsholme P., Gaudel C., Krause M. (2012). Mitochondria and diabetes. An intriguing pathogenetic role. Adv. Mitochon. Med..

[11] Sharifi-Rad M., Anil Kumar N.V., Zucca P., Varoni E.M., Dini L., Panzarini E., Rajkovic J., TsouhFokou P.V., Azzini E., Peluso I. (2020). Lifestyle, oxidative stress, and antioxidants: Back and forth in the pathophysiology of chronic diseases. Front. Physiol..

[12] Valko M., Leibfritz D., Moncol J., Cronin M.T., Mazur M., Telser J. (2007). Free radicals and antioxidants in normal physiological functions and human disease. Int. J. Biochem. Cell Biol..

[13] Brand M.D. (2010). The sites and topology of mitochondrial superoxide production. Exp. Gerontol..

[14] Vásquez-Vivar J., Kalyanaraman B., Kennedy M.C. (2000). Mitochondrial aconitase is a source of hydroxyl radical: An electron spin resonance investigation. J. Biol. Chem..

[15] Okado-Matsumoto A., Fridovich I. (2001). Subcellular distribution of superoxide dismutases (SOD) in rat liver: Cu, Zn-SOD in mitochondria. J. Biol. Chem..

[16] Yasui H., Hayashi S., Sakurai H. (2005). Possible involvement of singlet oxygen species as multiple oxidants in p450 catalytic reactions. Drug Metab. Pharmacokinet..

[17] Omura T. (2006). Mitochondrial P450s. Chem. Biol. Interact..

[18] Zumárraga M., Andía I., Dávila R., Miller J.C., Friedhoff A.J. (2004). Expression in normal and in subjects with schizophrenia of a novel gene fragment originally isolated from monozygotic twins discordant for schizophrenia. Genet. Mol. Biol..

[19] Zhou Y., Tao L., Zhou X., Zuo Z., Gong J., Liu X., Zhou Y., Liu C., Sang N., Liu H. (2021). DHODH and cancer: Promising prospects to be explored. Cancer Metab..

[20] Hey-Mogensen M., Goncalves R.L., Orr A.L., Brand M.D. (2014). Production of superoxide/H_2_O_2_ by dihydroorotate dehydrogenase in rat skeletal muscle mitochondria. Free Radic. Biol. Med..

[21] Popugaeva E., Bezprozvanny I. (2013). Role of endoplasmic reticulum Ca^2+^ signaling in the pathogenesis of Alzheimer disease. Front. Mol. Neurosci..

[22] Zorov D.B., Juhaszova M., Sollott S.J. (2014). Mitochondrial reactive oxygen species (ROS) and ROS-induced ROS release. Physiol. Rev..

[23] Kaludercic N., Mialet-Perez J., Paolocci N., Parini A., Di Lisa F. (2014). Monoamine oxidases as sources of oxidants in the heart. J. Mol. Cell. Cardiol..

[24] Rabilloud T., Heller M., Rigobello M.P., Bindoli A., Aebersold R., Lunardi J. (2001). The mitochondrial antioxidant defence system and its response to oxidative stress. Proteomics.

[25] Hanukoglu I. (2006). Antioxidant protective mechanisms against reactive oxygen species (ROS) generated by mitochondrial P450 systems in steroidogenic cells. Drug Metab. Rev..

[26] Rohan Fernando M., Lechner J.M., Löfgren S., Gladyshev V.N., Lou M.F., Rohan Fernando M., Lechner J.M., Löfgren S., Gladyshev V.N., Lou M.F. (2006). Mitochondrial thioltransferase (glutaredoxin 2) has GSH-dependent and thioredoxin reductase-dependent peroxidase activities in vitro and in lens epithelial cells. FASEB J..

[27] Kudryavtseva A.V., Krasnov G.S., Dmitriev A.A., Alekseev B.Y., Kardymon O.L., Sadritdinova A.F., Fedorova M.S., Pokrovsky A.V., Melnikova N.V., Kaprin A.D. (2016). Mitochondrial dysfunction and oxidative stress in aging and cancer. Oncotarget.

[28] Kirkinezos I.G., Moraes C.T. (2001). Reactive oxygen species and mitochondrial diseases. Semin. Cell Dev. Biol..

[29] Rimessi A., Previati M., Nigro F., Wieckowski M.R., Pinton P. (2016). Mitochondrial reactive oxygen species and inflammation: Molecular mechanisms, diseases and promising therapies. Int. J. Biochem. Cell Biol..

[30] Reshi M.S., Yadav D., Uthra C., Shrivastava S., Shukla S. (2020). Acetaminophen-induced renal toxicity: Preventive effect of silver nanoparticles. Toxicol. Res..

[31] Księżakowska-Łakoma K., Żyła M., Wilczyński J.R. (2014). Mitochondrial dysfunction in cancer. Prz. = Menopause Rev..

[32] Zou Z., Chang H., Li H., Wang S. (2017). Induction of reactive oxygen species: An emerging approach for cancer therapy. Apoptosis.

[33] Stadtman E.R., Levine R.L. (2000). Protein oxidation. Ann. N. Y. Acad. Sci..

[34] Fruhwirth G.O., Hermetter A. (2008). Mediation of apoptosis by oxidized phospholipids. Lipids Health Dis..

[35] Auten R.L., Whorton M.H., Nicholas Mason S. (2002). Blocking neutrophil influx reduces DNA damage in hyperoxia-exposed newborn rat lung. Am. J. Respir. Cell Mol. Biol..

[36] Parinandi N.L., Kleinberg M.A., Usatyuk P.V., Cummings R.J., Pennathur A., Cardounel A.J., Zweier J.L., Garcia J.G., Natarajan V. (2003). Hyperoxia-induced NAD (P) H oxidase activation and regulation by MAP kinases in human lung endothelial cells. Am. J. Physiol. Lung Cell Mol. Physiol..

[37] Lei K., Davis R.J. (2003). JNK phosphorylation of Bim-related members of the BCL2 family induces Bax-dependent apoptosis. Proc. Natl. Acad. Sci. USA.

[38] Di Marzo N., Chisci E., Giovannoni R. (2018). The role of hydrogen peroxide in redox-dependent signaling: Homeostatic and pathological responses in mammalian cells. Cells.

[39] Linley E., Denyer S.P., McDonnell G., Simons C., Maillard J.-Y. (2012). Use of hydrogen peroxide as a biocide: New consideration of its mechanisms of biocidal action. J. Antimicrob. Chemother..

[40] Jones D.P., Sies H. (2015). The Redox Code. Antioxid. Redox Signal..

[41] Sies H. (2017). Hydrogen peroxide as a central redox signaling molecule in physiological oxidative stress: Oxidative eustress. Redox Biol..

[42] Lorenzen I., Mullen L., Bekeschus S., Hanschmann E.-M. (2017). Redox regulation of inflammatory processes is enzymatically controlled. Oxid. Med. Cell. Longev..

[43] Veal E.A., Day A.M., Morgan B.A. (2007). Hydrogen peroxide sensing and signaling. Mol. Cell.

[44] Bienert G.P., Møller A.L.B., Kristiansen K.A., Schulz A., Møller I.M., Schjoerring J.K., Jahn T.P. (2007). Specific aquaporins facilitate the diffusion of hydrogen peroxide across membranes. J. Biol. Chem..

[45] Marinho H.S., Real C., Cyrne L., Soares H., Antunes F. (2014). Hydrogen peroxide sensing, signaling and regulation of transcription factors. Redox Biol..

[46] Lennicke C., Rahn J., Lichtenfels R., Wessjohann L.A., Seliger B. (2015). Hydrogen peroxide- production, fate and role in redox signaling of tumor cells. Cell Commun. Signal..

[47] Holmström K.M., Finkel T. (2014). Cellular mechanisms and physiological consequences of redox-dependent signalling. Nat. Rev. Mol. Cell Biol..

[48] Wang Y., Branicky R., Noë A., Hekimi S. (2018). Superoxide dismutases: Dual roles in controlling ROS damage and regulating ROS signaling. J. Cell Biol..

[49] Miller A.-F. (2012). Superoxide dismutases: Ancient enzymes and new insights. FEBS Lett..

[50] Sumimoto H., Miyano K., Takeya R. (2005). Molecular composition and regulation of the Nox family NAD(P)H oxidases. Biochem. Biophys. Res. Commun..

[51] Bedard K., Krause K.-H. (2007). The NOX family of ROS-generating NADPH oxidases: Physiology and pathophysiology. Physiol. Rev..

[52] Panday A., Sahoo M.K., Osorio D., Batra S. (2015). NADPH oxidases: An overview from structure to innate immunity-associated pathologies. Cell. Mol. Immunol..

[53] Miller E.W., Dickinson B.C., Chang C.J. (2010). Aquaporin-3 mediates hydrogen peroxide uptake to regulate downstream intracellular signaling. Proc. Natl. Acad. Sci. USA.

[54] Redza-Dutordoir M., Averill-Bates D.A. (2016). Activation of apoptosis signalling pathways by reactive oxygen species. Biochim. Biophys. Acta. Mol. Cell Res..

[55] Willcox J.K., Ash S.L., Catignani G.L. (2004). Antioxidants and prevention of chronic disease. Crit. Rev. Food Sci. Nutr..

[56] Pacher P., Beckman J.S., Liaudet L. (2007). Nitric oxide and peroxynitrite in health and disease. Physiol. Rev..

[57] Genestra M. (2007). Oxyl radicals, redox-sensitive signalling cascades and antioxidants. Cell. Signal..

[58] Halliwell B. (2007). Biochemistry of oxidative stress. Biochem. Soc. Trans..

[59] Young I.S., Woodside J.V. (2001). Antioxidants in health and disease. J. Clin. Pathol..

[60] Han R.N., Stewart D.J. (2006). Defective lung vascular development in endothelial nitric oxide synthase-deficient mice. Trends Cardiovasc. Med..

[61] Frei B. (1994). Reactive oxygen species and antioxidant vitamins. Am. J. Med..

[62] Nishida N., Arizumi T., Takita M., Kitai S., Yada N., Hagiwara S., Inoue T., Minami Y., Ueshima K., Sakurai T. (2013). Reactive oxygen species induce epigenetic instability through the formation of 8-hydroxydeoxyguanosine in human hepatocarcinogenesis. Dig. Dis..

[63] Yasui M., Kanemaru Y., Kamoshita N., Suzuki T., Arakawa T., Honma M. (2014). Tracing the fates of site-specifically introduced DNA adducts in the human genome. DNA Repair.

[64] Pizzino G., Irrera N., Cucinotta M., Pallio G., Mannino F., Arcoraci V., Squadrito F., Altavilla D., Bitto A. (2017). Oxidative stress: Harms and benefits for human health. Oxid. Med. Cell. Longev..

[65] O’Hagan H.M., Wang W., Sen S., Shields C.D., Lee S.S., Zhang Y.W., Clements E.G., Cai Y., Van Neste L., Easwaran H. (2011). Oxidative damage targets complexes containing DNA methyltransferases, SIRT1, and polycomb members to promoter CpG Islands. Cancer Cell.

[66] Guo R.F., Ward P.A. (2007). Role of oxidants in lung injury during sepsis. Antioxid. Redox Signal..

[67] Hoshino Y., Mishima M. (2008). Redox-based therapeutics for lung diseases. Antioxid. Redox Signal..

[68] Mahajan A., Tandon V.R. (2004). Antioxidants and rheumatoid arthritis. J. Indian Rheumatol. Assoc..

[69] Allan Butterfield D. (2002). Amyloid β-peptide (1-42)-induced oxidative stress and neurotoxicity: Implications for neurodegeneration in Alzheimer’s disease brain. A review. Free Radic. Res..

[70] Rai S., Hajam Y.A., Basheer M., Ghosh H. (2016). Biochemical and histopathological inflections in hepato-renal tissues of streptozotocin (STZ) induced diabetic male rats: Impact of exogenous melatonin administration. J. Clin. Res. Bioeth..

[71] Hajam Y.A., Rai S., Shree S., Basheer M., Ghosh H. (2017). Retrieval of reproductive complications by exogenous melatonin treatment in streptozotocin induced diabetic rat model. Res. Rev. J. Zool. Sci..

[72] Reichmann D., Voth W., Jakob U. (2018). Maintaining a healthy proteome during oxidative stress. Mol. Cell.

[73] Zhang J., Wang X., Vikash V., Ye Q., Wu D., Liu Y., Dong W. (2016). ROS and ROS-mediated cellular signaling. Oxid. Med. Cell. Longev..

[74] Sies H. (2015). Oxidative stress: A concept in redox biology and medicine. Redox Biol..

[75] Schieber M., Chandel N.S. (2014). ROS function in redox signaling and oxidative stress. Curr. Biol..

[76] Yatmaz S., Seow H.J., Gualano R.C., Wong Z.X., Stambas J., Selemidis S., Crack P.J., Bozinovski S., Anderson G.P., Vlahos R. (2013). Glutathione peroxidase-1 reduces influenza A virus–induced lung inflammation. Am. J. Respir. Cell Mol. Biol..

[77] Xu J., Li T., Wu H., Xu T. (2012). Role of thioredoxin in lung disease. Pulm. Pharmacol. Ther..

[78] Groitl B., Jakob U. (2014). Thiol-based redox switches. Biochim. Biophys. Acta Proteins Proteom..

[79] Garcia-Santamarina S., Boronat S., Hidalgo E. (2014). Reversible cysteine oxidation in hydrogen peroxide sensing and signal transduction. Biochemistry.

[80] Go Y.M., Jones D.P. (2010). Redox control systems in the nucleus: Mechanisms and functions. Antioxid. Redox Signal..

[81] Roos G., Messens J. (2011). Protein sulfenic acid formation: From cellular damage to redox regulation. Free Radic. Biol. Med..

[82] Corcoran A., Cotter T.G. (2013). Redox regulation of protein kinases. FEBS J..

[83] Östman A., Frijhoff J., Sandin Å., Böhmer F.D. (2011). Regulation of protein tyrosine phosphatases by reversible oxidation. J. Biochem..

[84] Adam-Vizi V., Chinopoulos C. (2006). Bioenergetics and the formation of mitochondrial reactive oxygen species. Trends Pharmacol. Sci..

[85] Song H., Bao S., Ramanadham S., Turk J. (2006). Effects of biological oxidants on the catalytic activity and structure of group VIA phospholipase A2. Biochem..

[86] Hajam Y.A., Rai S., Ghosh H., Basheer M. (2020). Combined administration of exogenous melatonin and insulin ameliorates streptozotocin induced toxic alteration on hematological parameters in diabetic male Wistar rats. Toxicol. Rep..

[87] Uthra C., Shrivastava S., Jaswal A., Sinha N., Reshi M.S., Shukla S. (2017). Therapeutic potential of quercetin against acrylamide induced toxicity in rats. Biomed. Pharmacother..

[88] Lobo V., Patil A., Phatak A., Chandra N. (2010). Free radicals, antioxidants and functional foods: Impact on human health. Pharmacogn. Rev..

[89] Birben E., Sahiner U.M., Sackesen C., Erzurum S., Kalayci O. (2012). Oxidative stress and antioxidant defense. World Allergy Organ J..

[90] Pham-Huy L.A., He H., Pham-Huy C. (2008). Free radicals, antioxidants in disease and health. Int. J. Biomed..

[91] Valavanidis A., Vlachogianni T., Fiotakis K., Loridas S. (2013). Pulmonary oxidative stress, inflammation and cancer: Respirable particulate matter, fibrous dusts and ozone as major causes of lung carcinogenesis through reactive oxygen species mechanisms. Int. J. Environ. Res. Public Health.

[92] Maechler P., Wollheim C.B. (2001). Mitochondrial function in normal and diabetic β-cells. Nature.

[93] Hastoy B., Godazgar M., Clark A., Nylander V., Spiliotis I., van de Bunt M., Chibalina M.V., Barrett A., Burrows C., Tarasov A.I. (2018). Electrophysiological properties of human beta-cell lines EndoC-βH1 and-βH2 conform with human beta-cells. Sci. Rep..

[94] Rustenbeck I., Schulze T., Morsi M., Alshafei M., Panten U. (2021). What is the metabolic amplification of insulin secretion and is it (still) relevant?. Metabolites.

[95] Cerf M.E. (2013). Beta cell dysfunction and insulin resistance. Front. Endocrinol..

[96] Silva J.P., Köhler M., Graff C., Oldfors A., Magnuson M.A., Berggren P.O., Larsson N.G. (2000). Impaired insulin secretion and β-cell loss in tissue-specific knockout mice with mitochondrial diabetes. Nat. Genet..

[97] Chinnery P.F. (2015). Mitochondrial disease in adults: What’s old and what’s new?. EMBO Mol. Med..

[98] Bhatti J.S., Bhatti G.K., Reddy P.H. (2017). Mitochondrial dysfunction and oxidative stress in metabolic disorders—A step towards mitochondria based therapeutic strategies. Biochim. Biophys. Acta Mol. Basis Dis..

[99] Sergi D., Naumovski N., Heilbronn L.K., Abeywardena M., O’Callaghan N., Lionetti L., Luscombe-Marsh N. (2019). Mitochondrial (Dys) function and insulin resistance: From pathophysiological molecular mechanisms to the impact of diet. Front. Physiol..

[100] Alfadda A.A., Sallam R.M. (2012). Reactive oxygen species in health and disease. J. Biomed. Biotechnol..

[101] He L., He T., Farrar S., Ji L., Liu T., Ma X. (2017). Antioxidants maintain cellular redox homeostasis by elimination of reactive oxygen species. Cell. Physiol. Biochem..

[102] Acharya J.D., Ghaskadbi S.S. (2010). Islets and their antioxidant defense. Islets.

[103] Chan C.B., De Leo D., Joseph J.W., McQuaid T.S., Ha X.F., Xu F., Tsushima R.G., Pennefather P.S., Salapatek A.M.F., Wheeler M.B. (2001). Increased uncoupling protein-2 levels in β-cells are associated with impaired glucose-stimulated insulin secretion: Mechanism of action. Diabetes.

[104] Zhu X.F., Zou H.D. (2012). PEDF in diabetic retinopathy: A protective effect of oxidative stress. J. Biomed. Biotechnol..

[105] Chen J.X., Stinnett A. (2008). Critical role of the NADPH oxidase subunit p47 phox on vascular TLR expression and neointimal lesion formation in high-fat diet-induced obesity. Lab. Investig..

[106] El-Haschimi K., Pierroz D.D., Hileman S.M., Bjorbaek C., Flier J.S. (2000). Two defects contribute to hypothalamic leptin resistance in mice with diet-induced obesity. J. Clin. Investig..

[107] Horvath T.L., Andrews Z.B., Diano S. (2009). Fuel utilization by hypothalamic neurons: Roles for ROS. Trends Endocrinol. Metab..

[108] Shih N.L., Cheng T.H., Loh S.H., Cheng P.Y., Wang D.L., Chen Y.S., Liu S.H., Liew C.C., Chen J.J. (2001). Reactive oxygen species modulate angiotensin II-induced β-myosin heavy chain gene expression via ras/raf/extracellular signal-regulated kinase pathway in neonatal rat cardiomyocytes. Biochem. Biophys. Res. Commun..

[109] Li J.M., Gall N.P., Grieve D.J., Chen M., Shah A.M. (2002). Activation of NADPH oxidase during progression of cardiac hypertrophy to failure. Hypertension.

[110] Satoh M., Ogita H., Takeshita K., Mukai Y., Kwiatkowski D.J., Liao J.K. (2006). Requirement of Rac1 in the development of cardiac hypertrophy. Proc. Natl. Acad. Sci. USA.

[111] Higuchi Y., Otsu K., Nishida K., Hirotani S., Nakayama H., Yamaguchi O., Hikoso S., Kashiwase K., Takeda T., Watanabe T. (2003). The small GTP-binding protein Rac1 induces cardiac myocyte hypertrophy through the activation of apoptosis signal-regulating kinase 1 and nuclear factor-κB. J. Biol. Chem..

[112] Izumiya Y., Kim S., Izumi Y., Yoshida K., Yoshiyama M., Matsuzawa A., Ichijo H., Iwao H. (2003). Apoptosis signal-regulating kinase 1 plays a pivotal role in angiotensin II–induced cardiac hypertrophy and remodeling. Circ. Res..

[113] Liu Q., Sargent M.A., York A.J., Molkentin J.D. (2009). ASK1 regulates cardiomyocyte death but not hypertrophy in transgenic mice. Circ. Res..

[114] Shanmugam P., Valente A.J., Prabhu S.D., Venkatesan B., Yoshida T., Delafontaine P., Chandrasekar B. (2011). Angiotensin-II type 1 receptor and NOX2 mediate TCF/LEF and CREB dependent WISP1 induction and cardiomyocyte hypertrophy. J. Mol. Cell. Cardiol..

[115] Mattson M.P. (2004). Pathways towards and away from Alzheimer’s disease. Nature.

[116] Andreyev A.Y., Kushnareva Y.E., Starkov A.A. (2005). Mitochondrial metabolism of reactive oxygen species. Biochem..

[117] Zhao Y., Zhao B. (2013). Oxidative stress and the pathogenesis of Alzheimer’s disease. Oxid. Med. Cell. Longev..

[118] Gandhi S., Abramov A.Y. (2012). Mechanism of oxidative stress in neurodegeneration. Oxid. Med. Cell. Longev..

[119] Radi E., Formichi P., Battisti C., Federico A. (2014). Apoptosis and oxidative stress in neurodegenerative diseases. J. Alzheimer’s Dis..

[120] Matsuoka Y., Picciano M., La Francois J., Duff K. (2001). Fibrillar β-amyloid evokes oxidative damage in a transgenic mouse model of Alzheimer’s disease. Neuroscience.

[121] Murakami K., Murata N., Noda Y., Tahara S., Kaneko T., Kinoshita N., Hatsuta H., Murayama S., Barnham K.J., Irie K. (2011). SOD1 (copper/zinc superoxide dismutase) deficiency drives amyloid β protein oligomerization and memory loss in mouse model of Alzheimer disease. J. Biol. Chem..

[122] Chen L., Na R., Gu M., Richardson A., Ran Q. (2008). Lipid peroxidation up-regulates BACE1 expression in vivo: A possible early event of amyloidogenesis in Alzheimer’s disease. J. Neurochem..

[123] Stamer K., Vogel R., Thies E., Mandelkow E., Mandelkow E.M. (2002). Tau blocks traffic of organelles, neurofilaments, and APP vesicles in neurons and enhances oxidative stress. J. Cell Biol..

[124] Yoshiyama Y., Higuchi M., Zhang B., Huang S.M., Iwata N., Saido T.C., Maeda J., Suhara T., Trojanowski J.Q., Lee V.M.Y. (2007). Synapse loss and microglial activation precede tangles in a P301S tauopathy mouse model. Neuron.

[125] David D.C., Hauptmann S., Scherping I., Schuessel K., Keil U., Rizzu P., Ravid R., Dröse S., Brandt U., Müller W.E. (2005). Proteomic and functional analyses reveal a mitochondrial dysfunction in P301L tau transgenic mice. J. Biol. Chem..

[126] Halverson R.A., Lewis J., Frausto S., Hutton M., Muma N.A. (2005). Tau protein is cross-linked by transglutaminase in P301L tau transgenic mice. J. Neurosci..

[127] Castellani R., Hirai K., Aliev G., Drew K.L., Nunomura A., Takeda A., Cash A.D., Obrenovich M.E., Perry G., Smith M.A. (2002). Role of mitochondrial dysfunction in Alzheimer’s disease. J. Neurosci. Res..

[128] Bubber P., Haroutunian V., Fisch G., Blass J.P., Gibson G.E. (2005). Mitochondrial abnormalities in Alzheimer brain: Mechanistic implications. Ann. Neurol..

[129] Wang X., Wang W., Li L., Perry G., Lee H.G., Zhu X. (2014). Oxidative stress and mitochondrial dysfunction in Alzheimer’s disease. Biochim. Biophys. Acta. Mol. Basis Dis..

[130] Moon H.E., Paek S.H. (2015). Mitochondrial dysfunction in Parkinson’s disease. Exp. Neurobiol..

[131] Blesa J., Trigo-Damas I., Quiroga-Varela A., Jackson-Lewis V.R. (2015). Oxidative stress and Parkinson’s disease. Front. Neuroanat..

[132] Schapira A.H. (2008). Mitochondria in the aetiology and pathogenesis of Parkinson’s disease. Lancet Neurol..

[133] Franco-Iborra S., Vila M., Perier C. (2016). The Parkinson disease mitochondrial hypothesis: Where are we at?. Neuroscientist.

[134] Lin M.T., Beal M.F. (2006). Mitochondrial dysfunction and oxidative stress in neurodegenerative diseases. Nature.

[135] Hauser D.N., Hastings T.G. (2013). Mitochondrial dysfunction and oxidative stress in Parkinson’s disease and monogenic parkinsonism. Neurobiol. Dis..

[136] Buneeva O., Fedchenko V., Kopylov A., Medvedev A. (2020). Mitochondrial dysfunction in Parkinson’s disease: Focus on Mitochondrial DNA. Biomedicines.

[137] Simon D.K., Pankratz N., Kissell D.K., Pauciulo M.W., Halter C.A., Rudolph A., Pfeiffer R.F., Nichols W.C., Foroud T. (2010). Maternal inheritance and mitochondrial DNA variants in familial Parkinson’s disease. BMC Med. Genet..

[138] Ellmore T.M., Suescun J., Castriotta R.J., Schiess M.C. (2020). A study of the relationship between uric acid and substantia nigra brain connectivity in patients with REM sleep behavior disorder and Parkinson’s disease. Front. Neurol..

[139] Ma L., Azad M.G., Dharmasivam M., Richardson V., Quinn R.J., Feng Y., Pountney D.L., Tonissen K.F., Mellick G.D., Yanatori I. (2021). Parkinson’s disease: Alterations in iron and redox biology as a key to unlock therapeutic strategies. Redox Biol..

[140] Oakley A.E., Collingwood J.F., Dobson J., Love G., Perrott H.R., Edwardson J.A., Elstner M., Morris C.M. (2007). Individual dopaminergic neurons show raised iron levels in Parkinson disease. Neurology.

[141] Locatelli F., Canaud B., Eckardt K.U., Stenvinkel P., Wanner C., Zoccali C. (2003). Oxidative stress in end-stage renal disease: An emerging threat to patient outcome. Nephrol. Dial. Transplant..

[142] Daenen K., Andries A., Mekahli D., Van Schepdael A., Jouret F., Bammens B. (2019). Oxidative stress in chronic kidney disease. Pediatr. Nephrol..

[143] Annuk M., Zilmer M., Lind L., Linde T., Fellström B. (2001). Oxidative stress and endothelial function in chronic renal failure. J. Am. Soc. Nephrol..

[144] Dounousi E., Papavasiliou E., Makedou A., Ioannou K., Katopodis K.P., Tselepis A., Siamopoulos K.C., Tsakiris D. (2006). Oxidative stress is progressively enhanced with advancing stages of CKD. Am. J. Kidney Dis..

[145] Ferraro B., Galli F., Frei B., Kingdon E., Canestrari F., Rice-Evans C., Buoncristiani U., Davenport A., Moore K.P. (2003). Peroxynitrite-induced oxidation of plasma lipids is enhanced in stable hemodialysis patients. Kidney Inter..

[146] Liakopoulos V., Roumeliotis S., Gorny X., Dounousi E., Mertens P.R. (2017). Oxidative stress in hemodialysis patients: A review of the literature. Oxid. Med. Cell. Longev..

[147] Krata N., Zagożdżon R., Foroncewicz B., Mucha K. (2018). Oxidative stress in kidney diseases: The cause or the consequence?. Arch. Immunol. Ther. Exp..

[148] Yang Q., Wu F.R., Wang J.N., Gao L., Jiang L., Li H.D., Ma Q., Liu X.Q., Wei B., Zhou L. (2018). Nox4 in renal diseases: An update. Free Radic. Biol. Med..

[149] Holterman C.E., Thibodeau J.F., Kennedy C.R. (2015). NADPH oxidase 5 and renal disease. Curr. Opin. Nephrol. Hypertens..

[150] Takac I., Schröder K., Zhang L., Lardy B., Anilkumar N., Lambeth J.D., Shah A.M., Morel F., Brandes R.P. (2011). The E-loop is involved in hydrogen peroxide formation by the NADPH oxidase Nox4. J. Biol. Chem..

[151] Monari A., Dumont E. (2015). Understanding DNA under oxidative stress and sensitization: The role of molecular modeling. Front. Chem..

[152] Descamps-Latscha B., Drüeke T., Witko-Sarsat V. (2001). Dialysis-induced oxidative stress: Biological aspects, clinical consequences, and therapy. Semin. Dial..

[153] Hajam Y.A., Rai S., Pandi-Perumal S.R., Brown G.M., Reiter R.J., Cardinali D.P. (2021). Coadministration of melatonin and insulin improves diabetic-induced impairment of rat kidney function. Neuroendocrinology.

[154] Zill H., Günther R., Erbersdobler H.F., Fölsch U.R., Faist V. (2001). RAGE expression and AGE-induced MAP kinase activation in Caco-2 cells. Biochem. Biophys. Res. Commun..

[155] Boulanger E., Wautier M.P., Wautier J.L., Boval B., Panis Y., Wernert N., Danze P.M., Dequiedt P. (2002). AGEs bind to mesothelial cells via RAGE and stimulate VCAM-1 expression. Kidney Inter..

[156] Ruiz S., Pergola P.E., Zager R.A., Vaziri N.D. (2013). Targeting the transcription factor Nrf2 to ameliorate oxidative stress and inflammation in chronic kidney disease. Kidney Inter..

[157] Reshi M.S., Shrivastava S., Jaswal A., Sinha N., Uthra C., Shukla S. (2017). Gold nanoparticles ameliorate acetaminophen induced hepato-renal injury in rats. Exp. Toxicol. Pathol..

[158] Modaresi A., Nafar M., Sahraei Z. (2015). Oxidative stress in chronic kidney disease. Iran. J. Kidney. Dis..

[159] Shokolenko I., Venediktova N., Bochkareva A., Wilson G.L., Alexeyev M.F. (2009). Oxidative stress induces degradation of mitochondrial DNA. Nucleic Acids Res..

[160] Adesso S., Popolo A., Bianco G., Sorrentino R., Pinto A., Autore G., Marzocco S. (2013). The uremic toxin indoxyl sulphate enhances macrophage response to LPS. PLoS ONE.

[161] Lopez-Otln C., Blasco M.A., Partridge L., Serrano M., Kroemer G. (2013). The hallmarks of aging. Cell.

[162] Benz C.C., Yau C. (2008). Ageing, oxidative stress and cancer: Paradigms in parallax. Nat. Rev. Cancer.

[163] Bonomini F., Rodella L.F., Rezzani R. (2015). Metabolic syndrome, aging and involvement of oxidative stress. Aging Dis..

[164] Liang R., Ghaffari S. (2014). Stem cells, redox signaling, and stem cell aging. Antioxid. Redox Signal..

[165] Martin J.E., Sheaff M.T. (2007). The pathology of ageing: Concepts and mechanisms. J. Pathol..

[166] Biala A.K., Dhingra R., Kirshenbaum L.A. (2015). Mitochondrial dynamics: Orchestrating the journey to advanced age. J. Mol. Cell. Cardiol..

[167] Bratic A., Larsson N.G. (2013). The role of mitochondria in aging. J. Clin. Investig..

[168] Indo H.P., Yen H.C., Nakanishi I., Matsumoto K.I., Tamura M., Nagano Y., Matsui H., Gusev O., Cornette R., Okuda T. (2015). A mitochondrial superoxide theory for oxidative stress diseases and aging. J. Clin. Biochem. Nutr..

[169] Genova M.L., Lenaz G. (2015). The interplay between respiratory supercomplexes and ROS in aging. Antioxid. Redox Signal..

[170] Barja G. (2014). The mitochondrial free radical theory of aging. Prog. Mol. Biol. Transl. Sci..

[171] López-Lluch G., Santos-Ocaña C., Sánchez-Alcázar J.A., Fernández-Ayala D.J.M., Asencio-Salcedo C., Rodríguez-Aguilera J.C., Navas P. (2015). Mitochondrial responsibility in ageing process: Innocent, suspect or guilty. Biogerontology.

[172] Bouzid M.A., Filaire E., McCall A., Fabre C. (2015). Radical oxygen species, exercise and aging: An update. Sports Med..

[173] Kwon M.J., Lee K.Y., Lee H.W., Kim J.H., Kim T.Y. (2015). SOD3 variant, R213G, altered SOD3 function, leading to ROS-mediated inflammation and damage in multiple organs of premature aging mice. Antioxid. Redox Signal..

[174] Edrey Y.H., Salmon A.B. (2014). Revisiting an age-old question regarding oxidative stress. Free Radic. Biol. Med..

[175] Bjelakovic G., Nikolova D., Gluud C. (2014). Antioxidant supplements and mortality. Curr. Opin. Clin. Nutr. Metab. Care.

[176] Cunningham G.M., Roman M.G., Flores L.C., Hubbard G.B., Salmon A.B., Zhang Y., Gelfond J., Ikeno Y. (2015). The paradoxical role of thioredoxin on oxidative stress and aging. Arch. Biochem. Biophys..

[177] Breitenbach M., Rinnerthaler M., Hartl J., Stincone A., Vowinckel J., Breitenbach-Koller H., Ralser M. (2014). Mitochondria in ageing: There is metabolism beyond the ROS. FEMS Yeast Res..

[178] Lagouge M., Larsson N.G. (2013). The role of mitochondrial DNA mutations and free radicals in disease and ageing. J. Intern. Med..

[179] Bertram C., Hass R. (2008). Cellular responses to reactive oxygen species-induced DNA damage and aging. Biol. Chem..

[180] Fimognari C. (2015). Role of oxidative RNA damage in chronic-degenerative diseases. Oxid. Med. Cell. Longev..

[181] Shimi T., Goldman R.D. (2014). Nuclear lamins and oxidative stress in cell proliferation and longevity. Can. Biol. Nucl. Envel..

[182] Rinnerthaler M., Bischof J., Streubel M.K., Trost A., Richter K. (2015). Oxidative stress in aging human skin. Biomolecules.

[183] Yan L.J. (2014). Positive oxidative stress in aging and aging-related disease tolerance. Redox Biol..

[184] Guillaumet-Adkins A., Yañez Y., Peris-Diaz M.D., Calabria I., Palanca-Ballester C., Sandoval J. (2017). Epigenetics and oxidative stress in aging. Oxid. Med. Cell. Longev..

[185] Area-Gomez E., Schon E.A. (2016). Mitochondria-associated ER membranes and Alzheimer disease. Curr. Opin. Genet. Dev..

[186] Area-Gomez E., Schon E.A. (2017). On the pathogenesis of Alzheimer’s Disease: The MAM hypothesis. FASEB J..

[187] Watanabe S., Ilieva H., Tamada H., Nomura H., Komine O., Endo F., Jin S., Mancias P., Kiyama H., Yamanaka K. (2016). Mitochondria-associated membrane collapse is a common pathomechanism in SIGMAR1- and SOD1-linked ALS. EMBO Mol. Med..

[188] Stoica R., De Vos K.J., Paillusion S., Mueller S., Sancho R.M., Lau K.-F., Vizcay-Barrena G., Lin W.-L., Xu Y.-F., Lewis J. (2014). ER-mitochondria associations are regulated by the VAPBPTPIP51 interaction and are disrupted by ALS/FTD-associated TDP-43. Nat. Commun..

[189] Stoica R., Paillusion S., Gomez-Suaga P., Mitchell J.C., Lau D.H., Gray E.H., Sancho R.M., Vizcay-Barrena G., De Vos K.J., Shaw C.E. (2016). ALS/FTD-associated FUS activates GSK-3beta to disrupt the VAPB-PTPIP51 interaction and ER-mitochondria associations. EMBO Rep..

[190] Tubbs E., Rieusset J. (2016). Metabolic signaling functions of ER-mitochondria contact sites: Role in metabolic diseases. Soc. Endocrinol..

[191] Tubbs E., Theurey P., Vial G., Bendridi N., Bravard A., Chauvin M.-A., Ji-Cao J., Zoulim F., Bartosch B., Ovize M. (2014). Mitochondria-associated endoplasmic reticulum membrane (MAM) integrity is required for insulin signaling and is implicated in hepatic insulin resistance. Diabetes.

[192] Arruda A.P., Pers B.M., Parlakgül G., Güney E., Inouye K., Hotamisligil G.S. (2014). Chronic enrichment of hepatic endoplasmic reticulummitochondria contact leads to mitochondrial dysfunction in obesity. Nat. Med..

[193] Sano R., Annunziata I., Patterson A., Moshiach S., Gomero E., Opferman J., Forte M., d’Azzo A. (2009). GM1-ganglioside accumulation at the mitochondria-associated ER membranes links ER stress to Ca(2+)-dependent mitochondrial apoptosis. Mol. Cell.

[194] Williamson C.D., Colberg-Poley A.M. (2009). Access of viral proteins to mitochondria via mitochondria-associated membranes. Rev. Med. Virol..

[195] English A.M., Schuler M.H., Xiao T., Kornmann B., Shaw J.M., Hughes A.L. (2020). ERmitochondria contacts promote mitochondrial-derived compartment biogenesis. J. Cell Biol..

[196] Goodrum J.M., Lever A.R., Coody T.K., Gottschling D.E., Hughes A.L. (2019). Rsp5 and Mdm30 reshape the mitochondrial network in response to age-induced vacuole stress. Mol. Biol. Cell..

[197] Schuler M.H., English A.M., Campbell T.J., Shaw J.M., Hughes A.L. (2020). Mitochondrialderived compartments facilitate cellular adaptation to amino acid stress. Mol. Cell.

[198] Chen Q., Samidurai A., Thompson J., Hu Y., Das A., Willard B., Lesnefsky E.J. (2020). Endoplasmic reticulum stress-mediated mitochondrial dysfunction in aged hearts. Biochim. Biophys. Acta Mol. Basis Dis..

[199] Bravo R., Vicencio J.M., Parra V., Troncoso R., Munoz J.P., Bui M., Lavandero S. (2011). Increased ER–mitochondrial coupling promotes mitochondrial respiration and bioenergetics during early phases of ER stress. J. Cell Sci..

[200] Glancy B., Balaban R.S. (2012). Role of mitochondrial Ca^2+^ in the regulation of cellular energetics. Biochemistry.

[201] Honrath B., Metz I., Bendridi N., Rieusset J., Culmsee C., Dolga A.M. (2017). Glucoseregulated protein 75 determines ER-mitochondrial coupling and sensitivity to oxidative stress in neuronal cells. Cell Death Discov..

[202] Liu X., Hajnoczky G. (2009). Ca^2+^-dependent regulation of mitochondrial dynamics by the Miro-Milton complex. Int. J. Biochem. Cell Biol..

[203] Knupp J., Arvan P., Chang A. (2019). Increased mitochondrial respiration promotes survival from endoplasmic reticulum stress. Cell Death Differ..

[204] Yang S., Zhou R., Zhang C., He S., Su Z. (2020). Mitochondria-associated endoplasmic reticulum membranes in the pathogenesis of type 2 diabetes mellitus. Front. Cell Dev. Biol..

[205] Conklin K.A. (2004). Chemotherapy-associated oxidative stress: Impact on chemotherapeutic effectiveness. Integr. Cancer Ther..

[206] Marullo R., Werner E., Degtyareva N., Moore B., Altavilla G., Ramalingam S.S., Doetsch P.W. (2013). Cisplatin induces a mitochondrial-ROS response that contributes to cytotoxicity depending on mitochondrial redox status and bioenergetic functions. PLoS ONE.

[207] Salehi F., Behboudi H., Kavoosi G., Ardestani S.K. (2018). Oxidative DNA damage induced by ROS-modulating agents with the ability to target DNA: A comparison of the biological characteristics of citrus pectin and apple pectin. Sci. Rep..

[208] Cannan W.J., Tsang B.P., Wallace S.S., Pederson D.S. (2014). Nucleosomes suppress the formation of double-strand DNA breaks during attempted base excision repair of clustered oxidative damages. J. Biochem..

[209] Curtin N.J. (2012). DNA repair dysregulation from cancer driver to therapeutic target. Nat. Rev. Cancer..

[210] Sancar A., Lindsey-Boltz L.A., Unsal-Kacmaz K., Linn S. (2004). Molecular mechanisms of mammalian DNA repair and the DNA damage checkpoints. Annu. Rev. Biochem..

[211] Goldstein M., Kastan M.B. (2015). The DNA damage response: Implications for tumor responses to radiation and chemotherapy. Annu. Rev. Med..

[212] Ciccia A., Elledge S.J. (2010). The DNA damage response: Making it safe to play with knives. Mol. Cell..

[213] McNally J.P., Millen S.H., Chaturvedi V., Lakes N., Terrell C.E., Elfers E.E., Carroll K.R., Hogan S.P., Andreassen P.R., Kanter J. (2017). Manipulating DNA damage-response signaling for the treatment of immune-mediated diseases. Proc. Natl. Acad. Sci. USA.

[214] Polo S.E., Jackson S.P. (2011). Dynamics of DNA damage response proteins at DNA breaks: A focus on protein modifications. Genes Dev..

[215] Elmore S. (2007). Apoptosis: A review of programmed cell death. Toxicol. Pathol..

[216] Maréchal A., Zou L. (2013). DNA damage sensing by the ATM and ATR kinases. Cold Spring Harb Perspect Biol..

[217] Altieri F., Grillo C., Maceroni M., Chichiarelli S. (2008). DNA damage and repair: From molecular mechanisms to health implications. Antioxid Redox Signal..

[218] Robertson A.B., Klungland A., Rognes T., Leiros I. (2009). DNA repair in mammalian cells: Base excision repair, the long and short of it. Cell Mol Life Sci..

